# *Candida albicans* Hxk1 influences expression of metabolic- and virulence-related genes

**DOI:** 10.1128/msphere.00395-25

**Published:** 2025-09-25

**Authors:** Stefanie Wijnants, Dimitrios Sofras, Wouter Van Genechten, Rudy Vergauwen, Ashley Valle Arevalo, Deepika Gunasekaran, Craig L. Ennis, Sabrina Jenull, Karl Kuchler, Clarissa J. Nobile, Patrick Van Dijck

**Affiliations:** 1 Laboratory of Molecular Cell Biology, Department of Biology, Institute of Botany and Microbiology, KU Leuven 118067 , Leuven, Belgium; 2 Department of Molecular and Cell Biology, School of Natural Sciences, University of California Merced 118577, Merced, California, USA; 3 Quantitative and Systems Biology Graduate Program, University of California 33244, Merced, California, USA; 4 Health Sciences Research Institute, University of California Merced 33244, Merced, California, USA; 5 Center for Medical Biochemistry, Max Perutz Labs Vienna, Campus Vienna Biocenter, Medical University of Vienna 27271, Vienna, Austria; 6 KU Leuven One Health Institute, Leuven, Belgium; University of Guelph, Guelph, Ontario, Canada

**Keywords:** *Candida albicans*, Hxk1, RNA-Seq, CUT&RUN, N-acetylglucosamine, metabolism, virulence

## Abstract

The Hxk1 protein of *Candida albicans* phosphorylates N-acetylglucosamine (GlcNAc) which is necessary for various cellular functions, including energy production and chitin synthesis. Further, this protein also regulates morphogenesis independently of its role in GlcNAc catabolism. When *HXK1* is deleted, cells are hyperfilamentous on serum-containing medium. Furthermore, Hxk1 translocates to the nucleus in the presence of glucose. To gain a broad understanding of the effect of Hxk1 on gene expression in *C. albicans*, we performed genome-wide transcriptional profiling of the *hxk1* mutant strain by RNA-Seq. The analysis of these RNA-Seq data showed that Hxk1 affects gene expression in both a carbon source-dependent and -independent manner. However, the effect on gene expression occurs via an indirect mechanism, as genome-wide CUT&RUN binding experiments demonstrated that Hxk1 does not bind to the upstream intergenic regions of the differentially expressed genes. Deletion of *HXK1* not only resulted in differences in gene expression of genes present in the GlcNAc and galactose regulons, but also in glucose transporter genes, including *HGT13*. Hxk1 also negatively influences the expression of virulence-associated genes, including *HWP1*, *BRG1*, and *UME6*. Consequently, an *hxk1* mutant strain showed higher toxicity toward gut epithelial cells compared to the WT strain. Furthermore, the *hxk1* mutant strain had higher expression levels of *SOD4* and *SOD5* and showed higher resistance toward H_2_O_2_. These findings highlight the multiple functions of Hxk1 in different cellular processes.

**IMPORTANCE:**

*Candida albicans* is a fungus that lives in the human body but does not cause any harm in healthy individuals. However, when the immune system is weakened, *C. albicans* can spread via the bloodstream all over the body and can lead to severe illness and even death. To infect the human body, multiple proteins hold distinct functions. Hxk1 is one of these proteins. This protein is involved in N-acetylglucosamine (GlcNAc) phosphorylation, as well as hyphae formation and glucose transport. To obtain a complete view of the processes regulated by Hxk1, we performed an RNA-Seq experiment. These data revealed that Hxk1 influences the regulation of genes involved in metabolic and virulence-related processes, such as GlcNAc metabolism, sterol metabolism, and oxidative stress resistance. These findings are important to better understand how *C. albicans* adapted itself to infect the host.

## INTRODUCTION

*Candida albicans* is an opportunistic human fungal pathogen that mostly lives as a commensal in the human host (1). Under favorable conditions, *C. albicans* becomes a pathogenic organism through the utilization of various virulence factors, including hyphal development, adhesion, biofilm formation, and secretion of hydrolytic enzymes (2). During its coevolution with the human host, several proteins acquired additional functions. Some of these proteins are involved in virulence, apart from their original function, which is often related to central metabolism. An example in this regard is Fba1, a glycolytic enzyme responsible for the conversion of fructose-1,6-bisphosphate into glyceraldehyde-3-phosphate and dihydroxy-acetone-phosphate. Remarkably, Fba1 is localized in the cell wall, where it binds to plasminogen, consequently enhancing the invasive potential of the fungus (3, 4). Another protein involved in the glycolytic pathway is Eno1, which is responsible for the conversion of 2-phosphoglycerate into phosphoenolpyruvate (5, 6). Eno1 is also present at the cell wall, where it can bind with human plasminogen to facilitate invasive infections (7–9). In addition, Eno1 also has transglutaminase activity (10). Furthermore, Tsa1 functioning depends on its localization. In hyphal cells, Tsa1 is localized to the cell wall and plays a role in the correct formation of the hyphal cell wall. On the other hand, in yeast cells, this protein is localized to the cytoplasm and is important for oxidative stress resistance (11).

Hxk1 exhibits dual cellular functionalities. First, Hxk1 phosphorylates N-acetylglucosamine (GlcNAc), which is the first step in the metabolic pathway catalyzing the use of this amino-sugar as a carbon and nitrogen source (12). Apart from this catabolic pathway, which is important when cells are growing inside macrophages (13), Hxk1 is also part of an anabolic pathway, where phosphorylated GlcNAc is a building block for the biosynthesis of the cell wall component chitin (14). In addition, this pathway is also important for the production of GPI-anchored proteins and proteins with an N-linked glycosyl group. The phosphatidylinositol (PI) components of these anchors are bound via a non-acetylated glucosamine to the mannose core (15). To use GlcNAc molecules during GPI anchor biosynthesis, GlcNAc needs to be phosphorylated by Hxk1 and converted through the anabolic pathway to UDP-GlcNAc (16). In the next step, the GlcNAc component is transferred from UDP-GlcNAc to PI to obtain GlcNAc-PI. This intermediate is converted into glucosaminyl phosphatidylinositol (GlcN-PI) (17). Second, Hxk1 also has regulatory functions. It is known that Hxk1 enters the nucleus in cells grown on low GlcNAc concentrations, glucose, or spider medium (containing mannitol as the carbon source) (18). This nuclear localization possibly influences specific gene expression, such as the regulation of *HXK2* and *GLK1/4*. Interestingly, the induction of GlcNAc-related genes seems to be independent of Hxk1 (19, 20). Furthermore, Hxk1 also affects the transport of glucose into the cells, as *hxk1* mutant strain shows an increased glucose transport (21). Remarkably, an *hxk1* mutant strain shows elevated expression of genes involved in the white–opaque epigenetic switch (18, 21–23). Furthermore, Hxk1 emerges as a morphogenesis regulator, distinct from its role in GlcNAc catabolism. *HXK1* is upregulated during hyphal conditions, which indicates its importance in filamentation. When *HXK1* is deleted, cells become hyperfilamentous on spider medium or on medium containing serum (18, 22). Notably, this modulation of filamentation occurs independently of known regulatory elements, such as Efg1, Ras1, Tup1, or Tpk1 (18). In contrast, another study showed that the *hxk1* mutant strain was able to form hyphae following stimulation with GlcNAc (19).

Taken together, Hxk1 exerts many functions in *C. albicans*. To gain a comprehensive view of the different roles played by this protein, we performed a genome-wide transcriptional profiling by RNA-Seq, combined with genome-wide binding by CUT&RUN, to determine whether Hxk1’s transcriptional responses are the result of a direct mechanism (i.e., Hxk1 functions itself as a transcription factor binding directly to downstream target genes) or an indirect mechanism (i.e., Hxk1 does not itself bind downstream target genes, but rather modulates their functions indirectly through transcription factors). Our RNA-Seq results indicate that the absence of Hxk1 influences the gene expression profiles of a broad spectrum of cellular processes. These processes include GlcNAc metabolism, galactose metabolism, glucose transport, and genes implicated in virulence pathways. Our investigation revealed that the effect of Hxk1 on gene expression is more pronounced when cells are grown on glycerol as opposed to glucose. A detailed exploration of the gene clusters exhibiting differential expression in the *hxk1* mutant strain has unveiled a carbon source-dependent influence of Hxk1 on the expression of GlcNAc-associated genes. Furthermore, Hxk1 has a negative effect on the gene expression of glucose transporter genes, including *HGT13*. Since Hxk1 negatively affects the expression of virulence-related genes, such as *HWP1*, *BRG1,* and *UME6*, the *hxk1* mutant strain showed a higher toxicity toward gut epithelial cells compared to the WT strain. Furthermore, the *hxk1* mutant strain had higher expression levels of *SOD4* and *SOD5*, resulting in higher resistance toward H_2_O_2_. These findings highlight the multifaceted functions of Hxk1 across a spectrum of cellular processes within *C. albicans* cells, where the effects seem to be the result of an indirect mechanism on gene expression.

## RESULTS

### Deletion of *HXK1* results in increased promoter activation of *HXK1*

**Fig 1:**
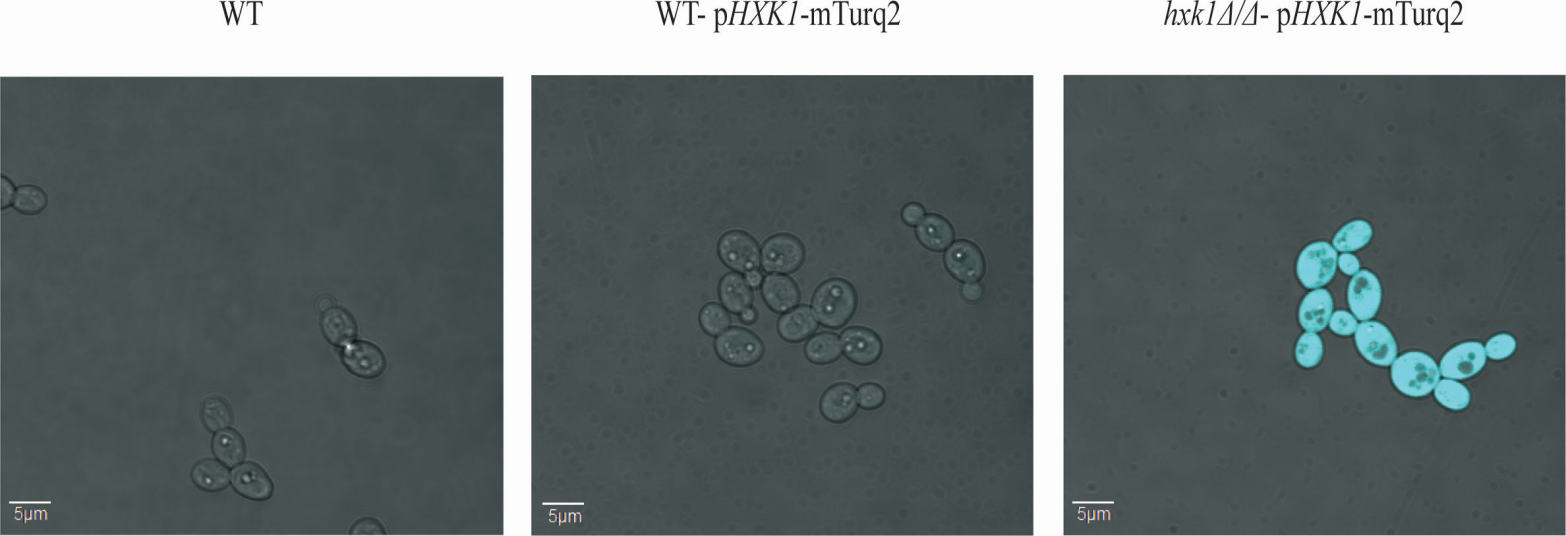
Deletion of *HXK1* results in higher activation of the *HXK1* promoter. *C. albicans* cells were exponentially grown in SC medium with 2% glucose and visually analyzed with a Fluoview FV1000 confocal scanning laser microscope. The mTurquoise2 fluorophore was excited with an argon laser at 458 nm, and emission was captured using a BA 480-495 bandpass filter. Twenty cells of each strain were imaged. This figure is a representation of two independent experiments each consisting of three biological repeats.

The regulation of genes participating in cellular metabolic processes is subject to precise control mechanisms (24). Given the prior demonstration of Hxk1’s regulatory influence on other metabolic-related genes, such as *HXK2*, *GLK1*, and *GLK4*, we investigated whether Hxk1 similarly governs its own expression (21). To explore this question, we introduced the *HXK1* promoter upstream of an mTurquoise2 fluorophore into the CIp10-mTurquoise2 plasmid (Table 1), which was subsequently transformed into both the WT and *hxk1* mutant strains (Table 2). Plasmid copy number validation was conducted to ensure a singular integration event in both strains. Subsequent examination of fluorescence was performed using confocal scanning laser microscopy. As illustrated in Fig. 1, the *hxk1* mutant strain exhibits increased fluorescence levels in contrast to the WT strain, indicating a higher activation of the *HXK1* promoter in the absence of *HXK1*.

**TABLE 1:** Plasmids used in this study

Plasmid	Genotype	Source
CIp10	p*ACT-NAT*-t*ACT*	
CIp10-*mTurq2*	p*ACT1-NAT*-t*ACT1* p*ACT1*-m*Turq2*-t*ACT1*	(25)
CIp10-p*HXK1-mTurq2*	p*ACT1-NAT*-t*ACT1* p*HXK1*-m*Turq2*-t*ACT1*	This work
CIp10-*HGT13*	p*ACT1-NAT*-t*ACT1* p*ACT1-HGT13*-t*ACT1*	This work

**TABLE 2:** *C. albicans* strains used in this study

Strains	Genotype	Source
SC5314		(26)
*hxk1D/D*	*hxk1D*::FRT*/hxk1D*::FRT	(21)
WT-p*HXK1*-mTurq2	*RPS1*/*RPS1*::(*pHXK1-mTurq2-tACT1*)	This work
*hxk1D/D*-p*HXK1*-mTurq2	*hxk1D*::FRT*/hxk1D*::FRT *RPS1*/*RPS1*::(*pHXK1-mTurq2-tACT1*)	This work
*HGT13^OE^*	*RPS1*/*RPS1*::(p*ACT1-HGT13*-t*ACT1*)	This work
WT-Hxk1-GFP	Hxk1-GFP	This work

### Hxk1 affects expression of both metabolic- and virulence-related genes

**Fig 2:**
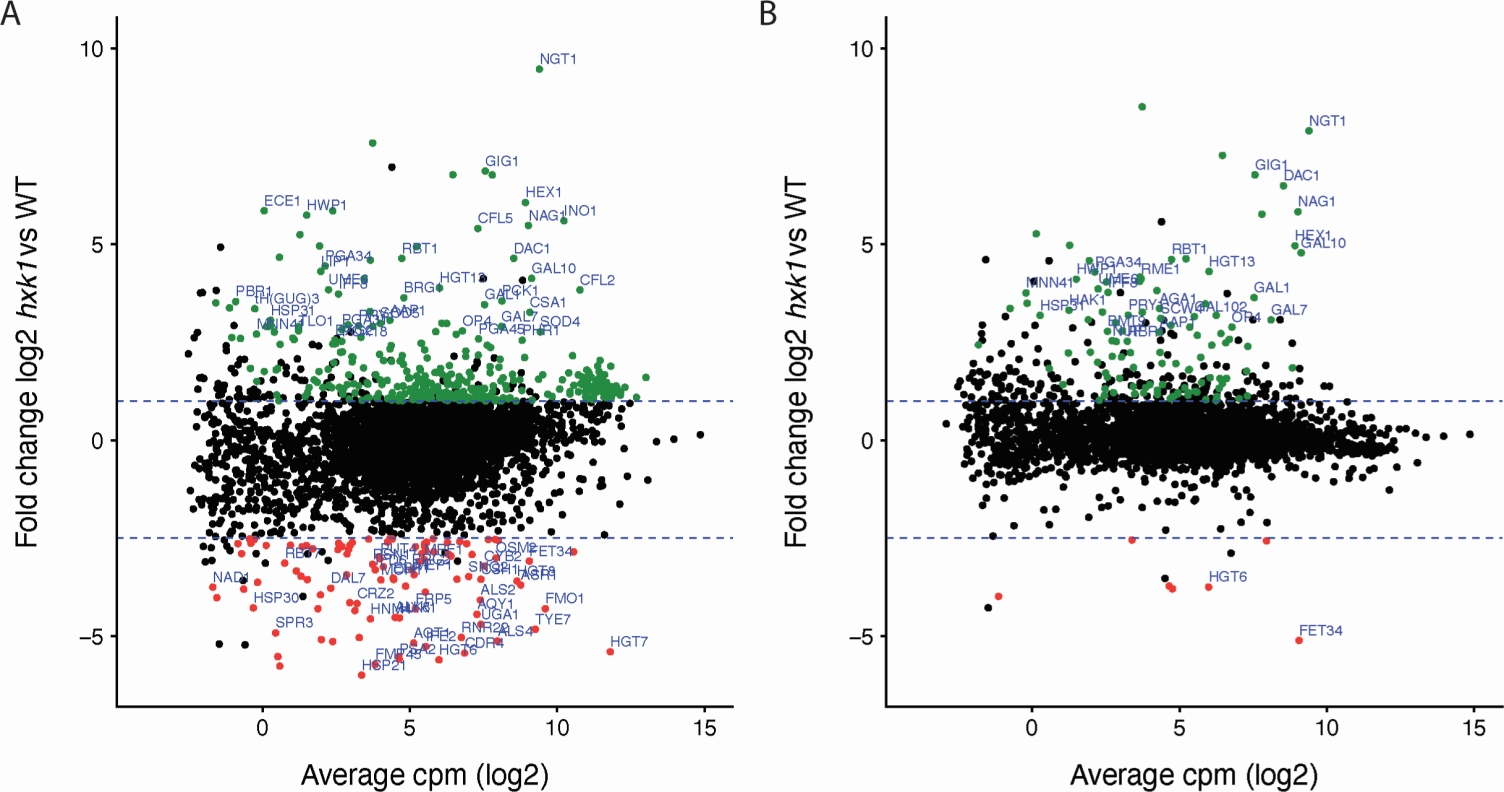
Hxk1 regulates the expression of both metabolic and virulence-related genes. (**A**) Differences in gene expression between the WT and *hxk1* mutant strains when cells were grown on 3% glycerol. (**B**) Differences in gene expression between the WT and *hxk1* mutant strains when cells were stimulated for 20 minutes with 2% glucose. The plots visualize the average log2 cpm (counts per million ((raw reads count of a gene/total reads in the sample) x 10^6^)) value (x-axis) against the log2-fold change in mRNA abundance between the average of two biological repeats of the WT and the *hxk1* mutant strains. Differentially expressed genes (FDR < 0.05) were displayed in green and red for upregulated and downregulated genes, respectively. The blue dashed lines highlight the log2-fold change of 1 and −2.5.

Hxk1 holds dual functionalities, involving both catalytic and regulatory roles, specifically GlcNAc phosphorylation and the regulation of *HXK2* and *GLK1/4* expression, respectively (12, 21). Previous research also elucidated the nuclear presence of Hxk1 in the context of glucose availability (18). Therefore, we explored the influence of Hxk1 on gene expression upon glucose addition. Using RNA-Seq, we mapped the gene expression profiles of both the WT and *hxk1* mutant strains before and after glucose addition (Fig. 2; Dataset S1 and S2). Initially, we hypothesized an effect of Hxk1 on gene expression in the presence of glucose, particularly due to prior indications of Hxk1’s nuclear translocation (18). We observed a few major differences in gene expression upon glucose addition between the WT and mutant strains, possibly due to *HXK1* repression by glucose.

In the presence of glycerol, we observed specific gene clusters, with genes involved in similar processes, that were differentially regulated in the *hxk1* mutant strain compared to the WT strain. First, genes encoding proteins involved in GlcNAc metabolism (*DAC1*, *NAG1*, *NGT1*, and *HEX1*) were upregulated in the *hxk1* mutant strain compared to the WT strain. This effect was also observed under glucose conditions, indicating a carbon source-independent effect on the regulation of these GlcNAc-related genes by Hxk1. Second, the genes of the *GAL* regulon (*GAL1*, *GAL7*, *GAL10*, and *GAL102*) were also upregulated in the *hxk1* mutant strain compared to the WT strain, both in the absence and presence of glucose. Furthermore, glucose transporter genes were differentially expressed in the *hxk1* mutant strain compared to the WT strain. The expression levels of *HGT6*, *HGT7*, and *HGT8* were downregulated. The expression of *HGT13* was upregulated in both the presence and absence of glucose. Genes encoding proteins involved in the ergosterol biosynthesis pathway (*ERG2*, *ERG3*, *ERG6*, *ERG10*, *ERG13*, and *ERG25*) were upregulated in the *hxk1* mutant strain compared to the WT strain. *SOD4* and *SOD5*, which encode proteins involved in the neutralization of reactive oxygen species (ROS) inside macrophages, showed an increased expression in the *hxk1* mutant strain (27). Finally, specific virulence-related genes were also differentially expressed in the *hxk1* mutant strain. *BRG1*, *LIP1*, *PRY1*, and *ECE1* were upregulated in the *hxk1* mutant strain in the absence of glucose, while *UME6* and *HWP1* showed an upregulated expression in both the absence and presence of glucose. The effect of Hxk1 on the regulation of these different gene clusters emphasizes its role in both metabolic- and virulence-related cellular processes.

### Hxk1 regulates genes involved in GlcNAc metabolism in a carbon source-dependent manner

**Fig 3:**
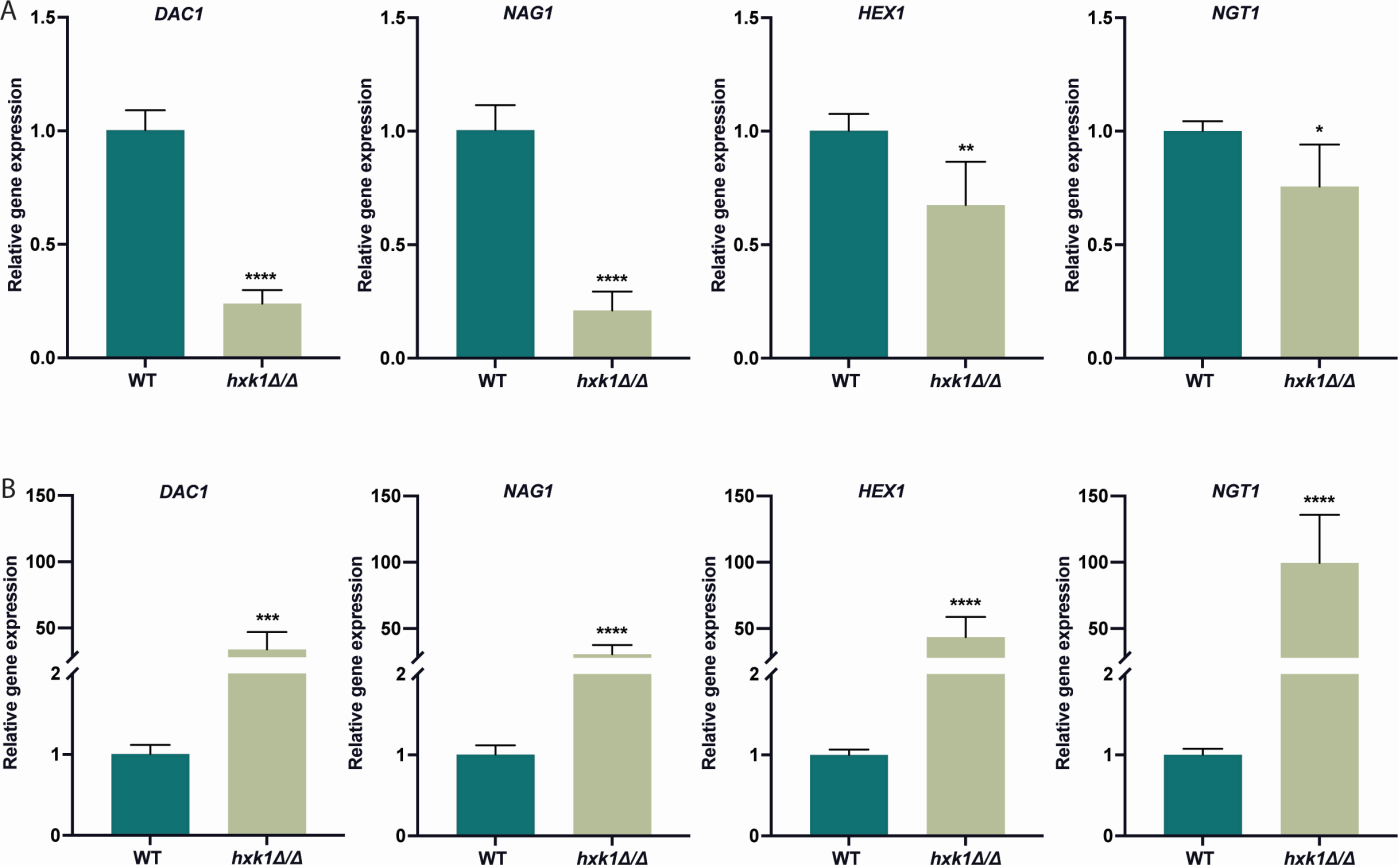
Hxk1 regulates the expression of genes involved in GlcNAc metabolism in a carbon source-specific manner. Cells were grown in SC medium with 3% glycerol until mid-exponential phase. Afterward, the cells were incubated for 20 minutes in the presence of either GlcNAc or galactose. qPCR was used to measure gene expression. (**A**) Expression levels of cells stimulated with GlcNAc. (**B**) Expression levels of cells stimulated with galactose. *ACT1*, *EFB1,* and *TEF1* were used as reference genes. The data are shown relative to the WT strain in the presence of GlcNAc or galactose. The average of two independent experiments each consisting of three biological and three technical repeats is shown, and the error bars represent the standard deviation (SD). Statistical analysis was done on log2(y)-transformed values using Student’s *t*-tests. Significant differences were indicated as *P* = 0.0332*, 0.0021**, 0.0002***, and ≤0.0001****.

The amino sugar GlcNAc, which is present in the human host’s mucosal layers and bacterial cell walls, can be used by *C. albicans* as a carbon and nitrogen source (28, 29). As mentioned above, different proteins play a role in the metabolism of this GlcNAc. The gene *HEX1* encodes a β-N-acetylglucosaminidase that degrades hyaluronic acid into GlcNAc (30, 31). These molecules can be transported into the cell via the GlcNAc-specific transporter Ngt1. This transporter is necessary for growth on low concentrations of GlcNAc (32). Inside the cell, GlcNAc can be converted into Fructose-6-phosphate during catabolic metabolism to gain energy. Dac1 and Nag1 are two enzymes involved in this process (33). Our RNA-Seq data showed that the expression of genes involved in GlcNAc metabolism (*DAC1*, *NAG1*, *HEX1*, and *NGT1*) was upregulated in the *hxk1* mutant strain compared to the WT strain for cells grown on both glycerol and glucose (Fig. 2). These results indicate that Hxk1 has a negative effect on the expression of these GlcNAc metabolism-related genes under these conditions. Because of these findings, we were interested to investigate the expression levels of these genes in the presence of other carbon sources (GlcNAc and galactose). We hypothesized that cells grown on galactose would exhibit the same expression pattern as those grown on glucose or glycerol. On the other hand, we hypothesized that for the cells grown on GlcNAc, the inhibiting effect of Hxk1 on gene expression would not be present as these genes have a function when this carbon source is available. Our results indeed showed a decreased expression of the genes involved in GlcNAc metabolism in the *hxk1* mutant strain compared to the WT strain in the presence of GlcNAc (Fig. 3A). This indicates that Hxk1 favors the expression of these genes in the presence of GlcNAc and confirms the carbon source-specific effect of Hxk1 on gene expression. On the contrary, when cells were grown on galactose, a gene expression pattern similar to that observed under glucose conditions was detected (Fig. 3B). This suggests an induction of GlcNAc-related genes in the *hxk1* deletion strain in the presence of GlcNAc and a repression of these genes when other carbon sources are present.

### Hxk1 influences the expression of different galactose-related genes

**Fig 4:**
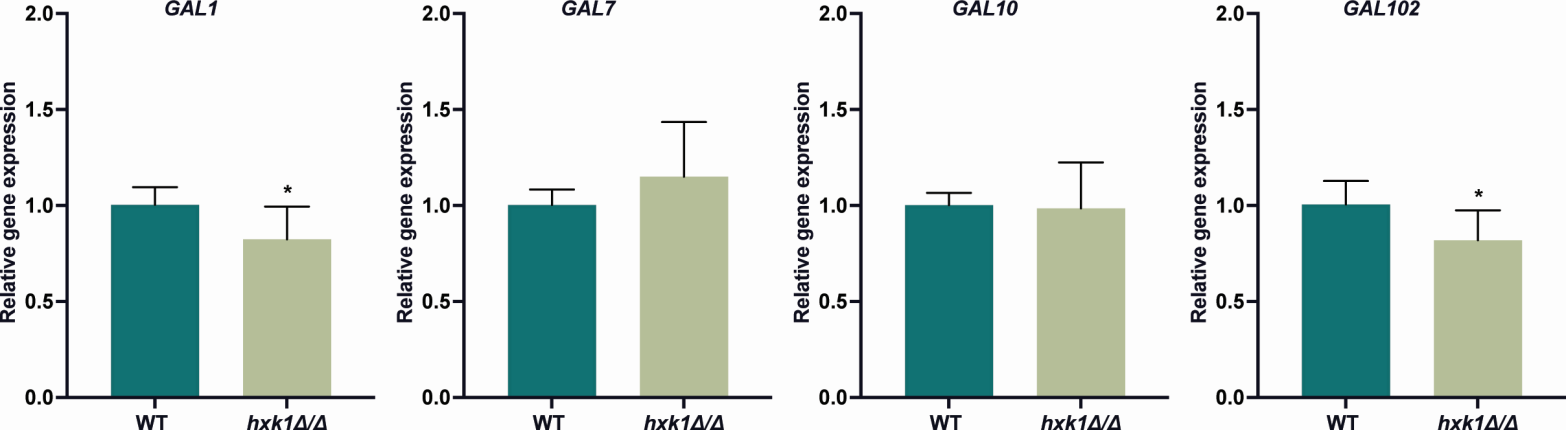
Hxk1 has both a positive and negative effect on the gene expression of the *GAL* genes in the presence of galactose. Cells were grown in SC medium with 3% glycerol until mid-exponential phase. Afterward, the cells were incubated for 20 minutes in the presence of 2% galactose. qPCR was used to measure gene expression. *18S*, *ACT1*, *EFB1,* and *TEF1* were used as reference genes. The data are shown relative to the WT strain in the presence of galactose. The average of two independent experiments each consisting of three biological and three technical repeats is shown, and the error bars represent the SD. Statistical analysis was done on log2(y)-transformed values using Student’s *t*-tests. A significant difference was indicated as *P = 0.0332.

*GAL1*, *GAL7*, *GAL10*, and *GAL102* are genes that are part of the *GAL* gene cluster. The first three genes are involved in galactose metabolism, while *GAL102* has a role in mannosylation of cell wall proteins (34–36). Hxk1 influences the expression of these genes both in the presence and absence of glucose (Fig. 2). Therefore, we wanted to investigate whether Hxk1 also plays a role when cells are grown on galactose, the substrate of the proteins encoded by these genes. Both *GAL1* and *GAL102* showed a decreased expression in the *hxk1* mutant strain compared to the WT strain in the presence of galactose (Fig. 4). Although the differences are significant, the magnitude of the differences is very small, suggesting that these differences are unlikely to result in phenotypic changes. *GAL7* and *GAL10* were not differentially expressed on galactose medium (Fig. 4).

### The increased glucose transport in the *hxk1* mutant strain is not caused by increased expression of *HGT13*

**Fig 5:**
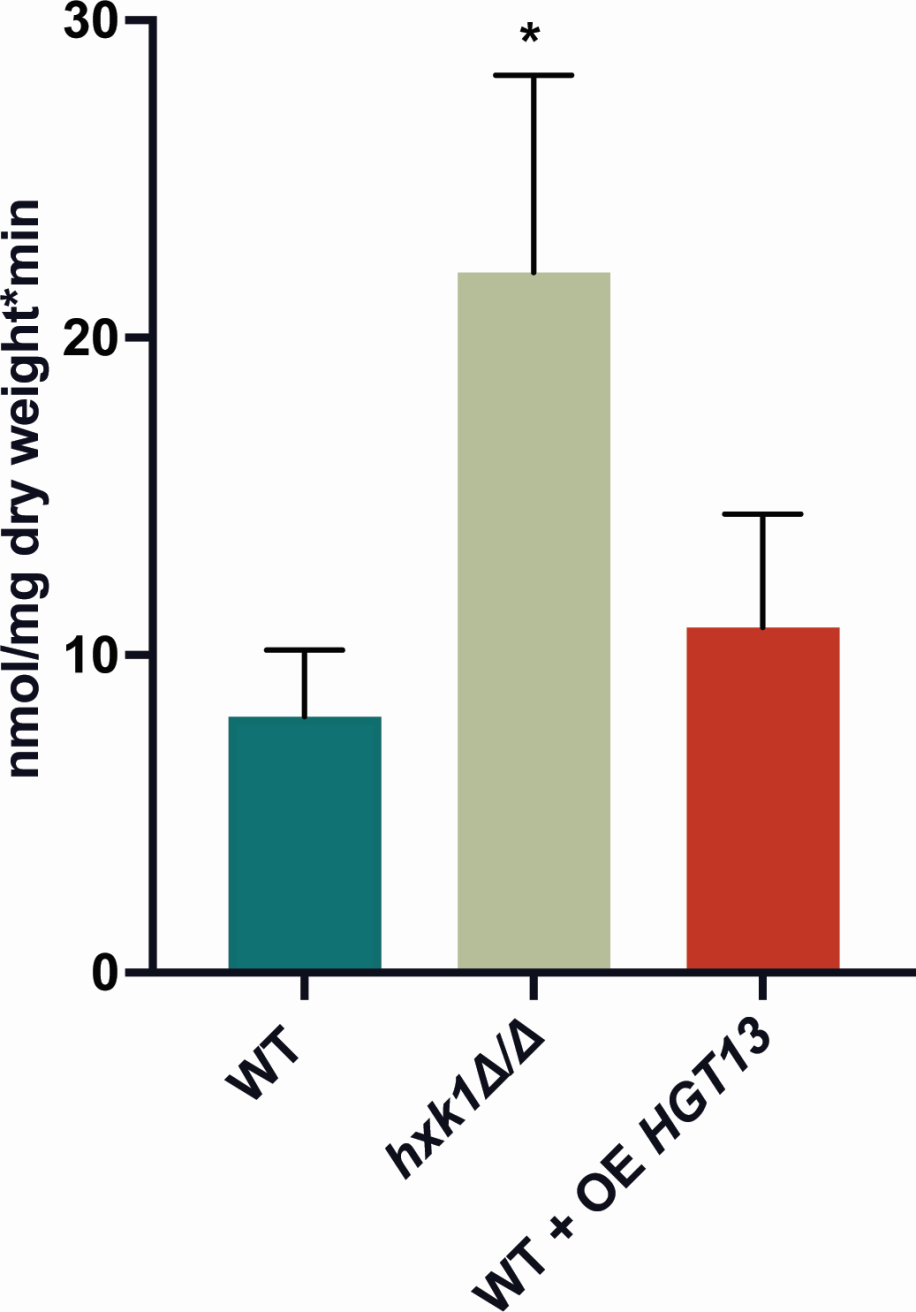
The increased glucose transport in the *hxk1* mutant strain is not caused by upregulation of *HGT13*. 2.5 mM of glucose suspension with ^14^C-labeled glucose (1500 cpm/nmol glucose) was added to the cells. The amount of radioactive glucose present in the cells was determined by measuring the radioactive levels by cpm (counts per minute). The average of three independent experiments each consisting of one biological and three technical repeats is shown, and the error bars represent the SD. Statistical analysis was done using an ANOVA test with Bonferroni correction. A significant difference was indicated as *P = 0.0332.

Previously, it was shown that glucose transport is upregulated in the *hxk1* mutant strain compared to the WT strain (21). Our RNA-Seq experiments revealed an increased expression of *HGT13* in the *hxk1* mutant strain (Fig. 2). This gene is involved in glucose transport (37). Therefore, we investigated if this increased glucose transport in the mutant strain is caused by an upregulation of *HGT13*. We generated an overexpression strain of *HGT13*, which was verified by qPCR (Fig. S1). However, during our transport studies, we did not detect an increased import of glucose into these overexpression strains (Fig. 5). This indicates that the higher amount of glucose transported in the *hxk1* mutant strain was achieved independently of the increased expression of *HGT13* in this strain.

### Differentially expressed genes involved in ergosterol biosynthesis cause alterations in sterol pattern

**Fig 6:**
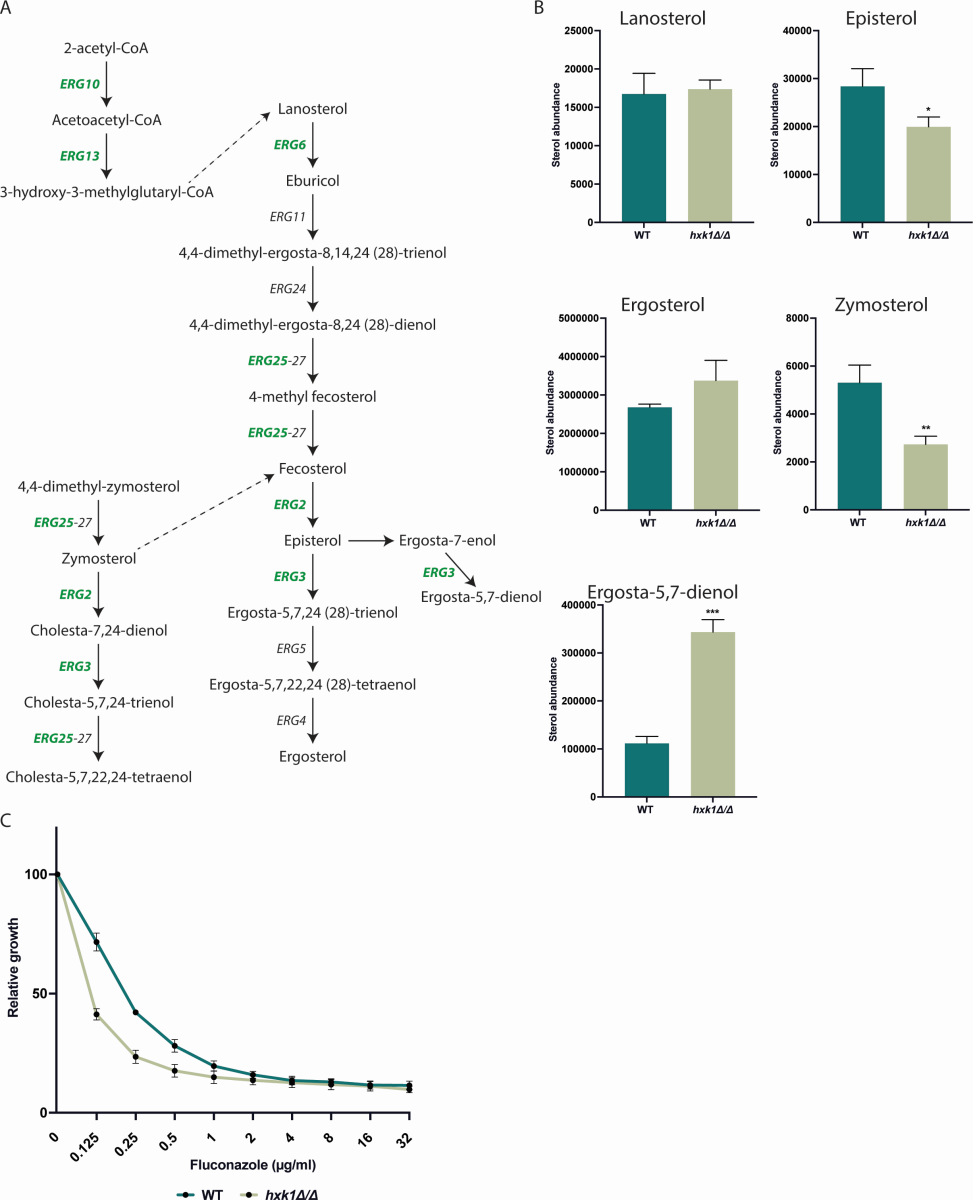
Upregulation of *ERG* genes in the *hxk1* mutant strain results in an altered sterol content in the cells. (**A**) Overview of sterol metabolism. The expression of the genes in green was found to be upregulated in the absence of glucose in the *hxk1* mutant strain. (**B**) The lanosterol, episterol, ergosterol, zymosterol, and ergosta-5,7-dienol content of the cells was measured by GC-MS. This figure shows the data of one experiment performed with three biological and two technical repeats with the error bars representing the SD. The result of this experiment was representative for the three performed experiments. Statistical analysis was done using Student’s *t*-tests and significant differences were indicated as *P* = 0.0332*, 0.0021**, and 0.0002***. (**C**) The resistance of the WT and *hxk1* mutant strains toward fluconazole was measured via a broth dilution assay. The average of two independent experiments each consisting of three biological and two technical repeats is shown, and the error bars represent the SD.

Sterols are important components of the cell membrane, making them compelling targets for antifungal drug development (38). The azoles target enzymes involved in the sterol biosynthesis pathway, while the polyenes target sterols in the plasma membrane itself (Fig. 6A). The data from our RNA-Seq experiment on glycerol medium showed that the *hxk1* mutant strain had increased expression levels of genes involved in sterol biosynthesis, namely *ERG2*, *ERG3*, *ERG6*, *ERG10*, *ERG13*, and *ERG25* (Fig. 2). We isolated the sterols of both the WT strain and the *hxk1* mutant strain grown on glycerol and analyzed the samples using gas chromatography-mass spectrometry (GC-MS). This data showed no significant difference in ergosterol content between the WT and the *hxk1* mutant strains but showed an increased level of ergosta-5,7-dienol in the *hxk1* mutant strain (Fig. 6B). On the other hand, levels of zymosterol and episterol were decreased in the *hxk1* mutant strain. Furthermore, no increased levels of toxic sterols were observed in the *hxk1* mutant strain (Fig. 6B) (39). To determine whether this shift in sterol intermediates influences the fluconazole resistance of *C. albicans*, we performed a broth dilution assay with different concentrations of fluconazole on glycerol-containing medium. The result from this experiment showed that the *hxk1* mutant strain is more susceptible to fluconazole with a minimal inhibitory concentration at 50% (MIC_50_) of 0.125 µg/mL, while the WT strain has an MIC_50_ of 0.25 µg/mL (Fig. 6C). This shows a correlation between the differences in ergosterol intermediates and changes in fluconazole susceptibility.

### The *hxk1* mutant strain has a higher tolerance toward H_2_O_2_

**Fig 7:**
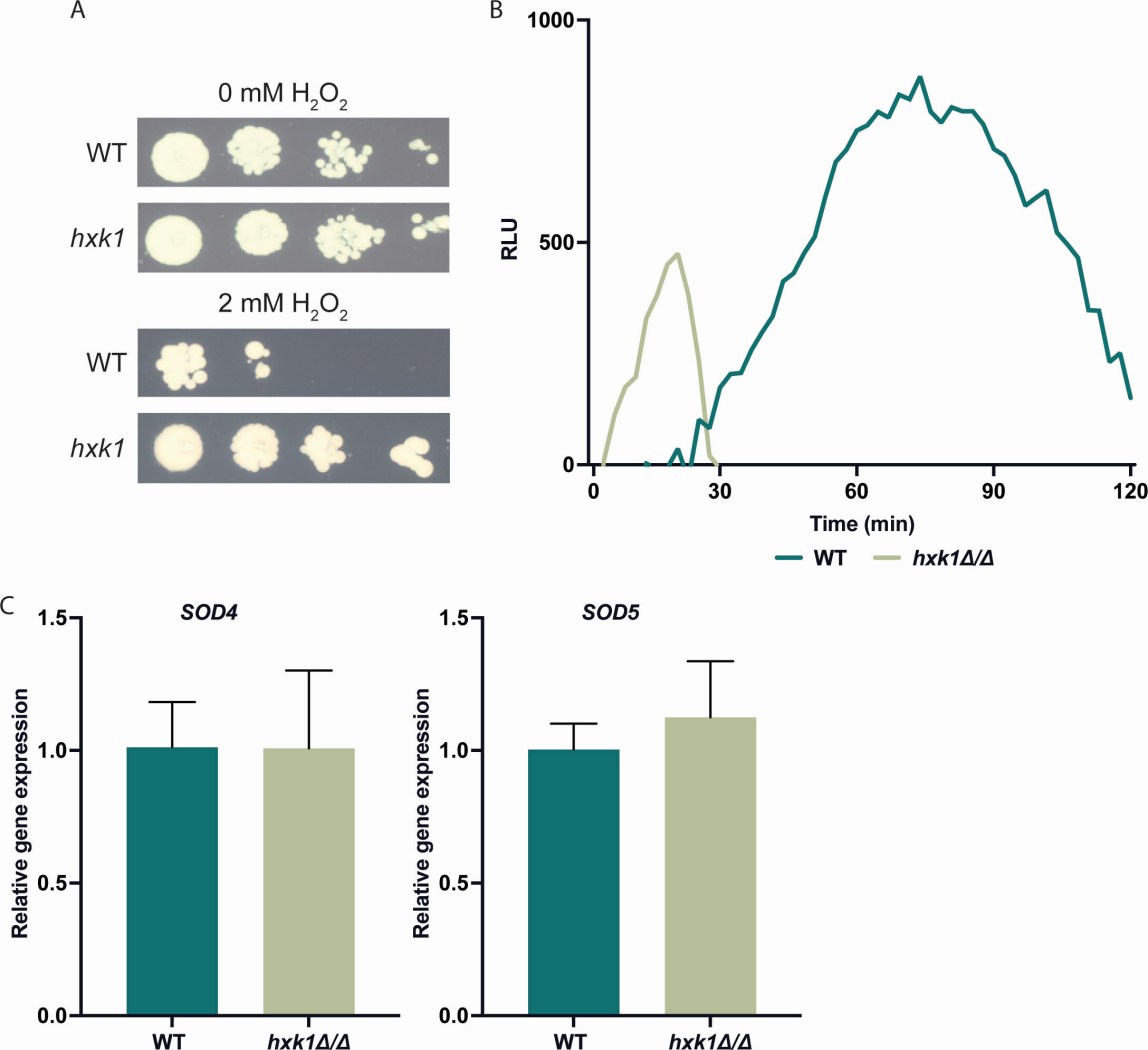
The *hxk1* mutant strain has an increased resistance to H_2_O_2_. (**A**) *C. albicans* cells were grown on SC (2% glucose) medium and SC (2% glucose) medium supplemented with 2 mM H_2_O_2_ to check their tolerance toward oxidative stress. This figure shows the data of one experiment performed with three biological and two technical repeats. The result of this experiment was representative of the three performed experiments. (**B**) BMDMs and *C. albicans* cells were co-cultured for 2 hours and the ROS molecules produced by the macrophages were measured over time. This graph shows the data of one experiment consisting of one biological repeat and three technical repeats. The result of this experiment was representative of the three performed experiments. (**C**) BMDMs and *C. albicans* cells were co-cultured for 3 hours, and fungal RNA was collected to measure gene expression. *18S*, *EFB1,* and *TEF1* were used as reference genes. The data are shown relative to the WT strain in the presence of macrophages. The average of two independent experiments consisting of three biological repeats and three technical repeats is shown, and the error bars represent the SD. Statistical analysis was done on log2(y)-transformed values using Student’s *t*-tests.

Inside immune cells, *C. albicans* can negatively affect the maturation of phagosomes and thereby prevent fungal cell killing (40). For example, *C. albicans* copes with toxic ROS chemicals, such as O_2_^-^, produced by macrophages during an oxidative burst (41). To counteract this burst, *C. albicans* decomposes these ROS molecules with the superoxide dismutases Sod4 and Sod5. *C. albicans* strains lacking these Sod proteins are more vulnerable to killing by phagosomes (27). Since the *hxk1* mutant strain shows an upregulation of *SOD4* and *SOD5* expression in the presence of glycerol, we wanted to investigate if this strain shows a higher tolerance toward ROS (Fig. 2). These SOD enzymes produce H_2_O_2_ molecules, so increased expression of *SOD* genes can lead to higher levels of H_2_O_2_ that need to be tolerated by the cells. Therefore, we tested the H_2_O_2_ tolerance of the WT and *hxk1* mutant strains in a spot assay (Fig. 7A). These data indicate a correlation between the increased expression of *SOD4* and *SOD5* and a higher tolerance toward H_2_O_2_.

Given the increased tolerance to H_2_O_2_ observed in the *hxk1* mutant strain under *in vitro* conditions, we sought to determine whether this mutant strain influenced ROS accumulation differently in macrophages. Therefore, we co-cultured macrophages and *C. albicans* cells in cell culture medium for 2 hours and measured ROS production by the macrophages to counteract the *C. albicans* cells. The results showed a different ROS accumulation pattern of macrophages infected with the WT and *hxk1* mutant strains (Fig. 7B). Macrophages infected with the WT strain showed a slow increase of ROS over time, while the macrophages infected with the *hxk1* mutant strain showed a rapid increase, followed by a rapid decrease of ROS. This latter observation is possibly due to a more rapid detoxification of the ROS by the *hxk1* mutant strain, as this strain upregulates *SOD4* and *SOD5*. However, when we examined the expression of *C. albicans SOD4* and *SOD5* during co-culture of the fungal cells with macrophages, we did not observe a difference in expression between the WT and the *hxk1* mutant strains (Fig. 7C). Therefore, we conclude that the difference in ROS accumulation by macrophages infected with the WT strain and *hxk1* mutant strain cells is not due to differences in the expression of *SOD4* and *SOD5*.

### Deletion of *HXK1* results in a higher toxicity toward gut epithelial cells

**Fig 8:**
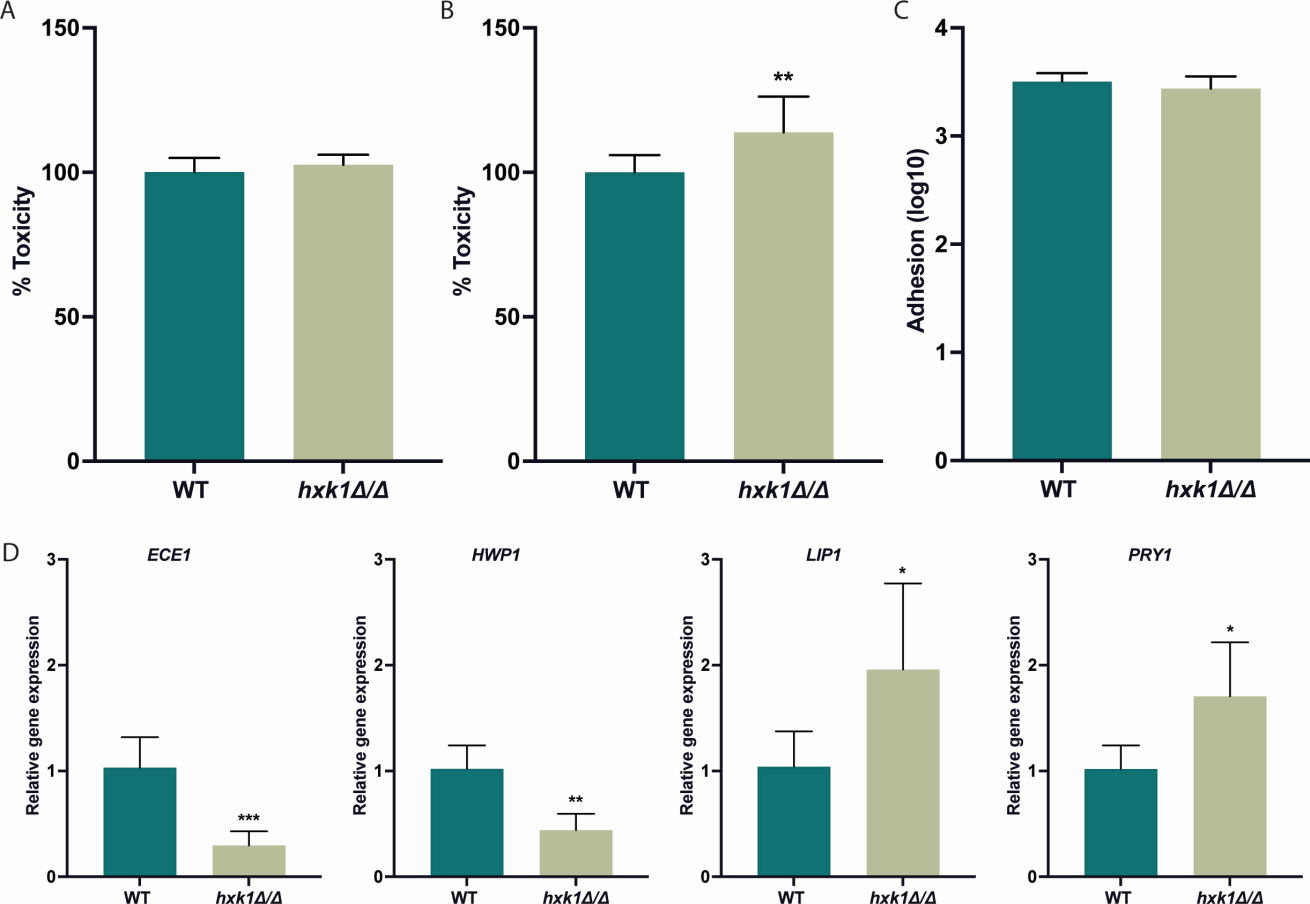
Deletion of *HXK1* results in an increased toxicity toward gut epithelial cells. (**A**) *C. albicans* cells were incubated for 24 hours with tongue epithelial cells (TR146), and toxicity was measured by checking LDH release. The levels of LDH release were normalized to the values of the WT, which gives the WT 100% toxicity. The average of two independent experiments consisting of three biological repeats and three technical repeats is shown, and the error bars represent the SD. (**B**) *C. albicans* cells were incubated for 24 hours with gut epithelial cells (CaCo2), and toxicity was measured by checking LDH release. The levels of LDH release were normalized to the values of the WT, which gives the WT 100% toxicity. The results show the average of three technical repeats. The average of two independent experiments consisting of three biological repeats and three technical repeats is shown, and the error bars represent the SD. (**C**) *C. albicans* cells were co-cultured with CaCo2 cells for 90 minutes to check their adhesion capacity. The average of two independent experiments consisting of three biological repeats and three technical repeats is shown, and the error bars represent the SD. (**D**) CaCo2 cells and *C. albicans* cells were co-cultured for 6 hours, and fungal RNA was collected to measure gene expression. *18S*, *EFB1,* and *TEF1* were used as reference genes. The data are shown relative to the WT strain in the presence of epithelial cells. The average of two independent experiments consisting of three biological repeats and three technical repeats is shown, and the error bars represent the SD. Statistical analysis was done on log2(y)-transformed values using Student’s *t*-tests and significant differences were indicated as *P* = 0.0332*, 0.0021**, and 0.0002***.

*C. albicans* penetrates through the host epithelial cell layer, enters the bloodstream, and causes a systemic infection. Different proteins are involved in this process, including Hwp1, Brg1, Ece1, Lip1, Ume6, and Pry1 (42). For example, Hwp1 is a cell wall adhesin important for host cell interactions, as it covalently binds to host epithelial cells (43, 44), while Lip1 and Ece1 are important for the penetration of the *C. albicans* hyphal tip into host epithelial cells (45, 46). The genes that encode these six proteins are differentially expressed in the WT strain compared to the *hxk1* mutant strain. *BRG1*, *LIP1*, *PRY1*, and *ECE1* are upregulated in the mutant strain when cells were grown on glycerol, while *UME6* and *HWP1* were upregulated in the presence of both glucose and glycerol (Fig. 2). Given these expression differences, we hypothesized that the *hxk1* mutant strain could have an increased toxicity toward epithelial cells. Therefore, we investigated the toxicity of the WT and *hxk1* mutant strains toward both oral epithelial and gut epithelial cells. Regarding toxicity toward oral epithelial cells, we did not observe a difference in toxicity of the WT strain compared to the mutant strain when cultured in cell culture medium (Fig. 8A). On the contrary, the *hxk1* mutant strain showed a significant increase in toxicity toward gut epithelial cells compared to the WT strain; however, the magnitude of increase was not high (Fig. 8B). However, no difference in adhesion capacity between the WT strain and the *hxk1* mutant strain was observed toward these gut epithelial cells (Fig. 8C). Next, we checked the expression of a selection of these genes of interest after coculturing *C. albicans* cells with gut epithelial cells. Upon coculturing, compared to the WT strain, the expression of *ECE1* and *HWP1* was downregulated in the *hxk1* mutant strain, while the expression of *LIP1* and *PRY1* was upregulated (Fig. 8D). This led us to hypothesize that the increased toxicity caused by the *hxk1* mutant strain toward gut epithelial cells is partially caused by an upregulation of *LIP1* and *PRY1*.

### Hxk1 influences gene expression via an indirect mechanism

The RNA-Seq results showed that Hxk1 influenced the expression of genes involved in different *C. albicans* cellular processes. However, it was still unclear whether Hxk1 was acting directly on its downstream target genes as a transcription factor or if a more complex indirect regulatory process was involved. To determine whether Hxk1 has a direct or indirect effect on gene expression, a CUT&RUN experiment was performed with Hxk1 GFP-tagged WT cells grown for 20 minutes on glucose—the same conditions as the RNA-seq experiment. All binding locations are listed in Dataset S3. This experiment demonstrated that Hxk1 bound to the upstream intergenic regions of 28 genes across the genome (see Dataset S4 for plots of the genomic regions bound upstream of these 28 genes), showing relatively low enrichment for binding enrichment typically observed for transcription factors by us and others. In addition, several of these Hxk1 binding events also had notable binding in the IgG control sample, suggesting that they could be false-positive binding events in “sticky” regions of the genome, despite being significantly called as peaks post-normalization to the IgG control. None of these 28 genes were differentially expressed in the RNA-Seq experiment when we compared the WT and *hxk1* mutant strains. We also note that none of the 28 genes encoded for known transcription factors. Since these few directly bound genes were not transcriptionally regulated and likely to be spurious, we conclude that Hxk1 does not act as a transcription factor to regulate the expression of its downstream target genes, but rather that Hxk1 has an indirect effect on gene expression through a mechanism that is currently unknown.

## DISCUSSION

With this study, we demonstrate the importance of Hxk1 on the expression of genes involved in different cellular processes in *C. albicans*. We performed RNA-Seq to compare the expression profiles of the WT strain and the *hxk1* mutant strain, both in the absence and presence of glucose. These data show us that deletion of *HXK1* results in a broad effect on gene expression in the absence of glucose compared to in the presence of glucose. Furthermore, deletion of *HXK1* seems to affect the expression of genes involved in both metabolic- and virulence-related processes. In addition, deletion of *HXK1* may lead to GlcNAc accumulation, which can trigger the GlcNAc signaling pathway and influence the regulation of various cellular processes.

As previously shown, Hxk1 regulates the expression of specific glucose phosphorylating kinases in *C. albicans* (21). Furthermore, deletion of *HXK1* results in an increased *HXK1* promoter activation. It is possible that this is due to the fact that Hxk1 exercises a negative feedback loop mechanism or that, due to the accumulation of GlcNAc in the medium, the GlcNAc pathway is overactivated and hyperactivates the *HXK1* promoter.

Given that Hxk1 translocates to the nucleus in the presence of glucose, it was expected to affect gene expression under glucose conditions; however, only minimal effects on gene expression were observed (18). It is possible that Hxk1 also translocates to the nucleus in the presence of glycerol to modulate gene expression. However, since *HXK1* expression is repressed by glucose, its impact on gene expression may be diminished under glucose conditions compared to glycerol conditions.

Hxk1 is well known for its role in phosphorylating GlcNAc, an alternative carbon source for *C. albicans*, and is essential for full virulence in the host (12). In addition, Hxk1 has a negative effect on the expression of the specific GlcNAc-related genes, such as *DAC1*, *NAG1*, *HEX1*, and *NGT1*, in the absence of GlcNAc. This repression may be associated with carbon catabolite inactivation in the presence of glucose. Although this process has been lost in *C. albi*cans compared to *S. cerevisiae*, genes involved in alternative metabolism are expected to be repressed in the presence of glucose (47).

The increased expression levels of the *GAL* genes in the *hxk1* mutant strain in the absence of galactose can be caused by different mechanisms. First, Hxk1 can inhibit *GAL* gene expression in the absence of a substrate. Alternatively, it has been reported in the literature that the expression of *GAL* genes is influenced by GlcNAc (48). Consequently, deletion of *HXK1* can result in the accumulation of GlcNAc and hence in the increased expression of *GAL*-related genes. The differences in expression of the *GAL* genes when grown on galactose are unexpected, as all these genes are part of the *GAL* regulon (34–36). We expected that the expression of *GAL1* would be decreased in the *hxk1* mutant strain when grown on galactose as the substrate for this enzyme is present. In addition, most of the galactose-induced genes are regulated via Rgt1 (49, 50). Thus, it is possible that Hxk1 regulates the expression of *GAL1* and *GAL102* via Rgt1. However, since only differences in expression were observed for these two genes in the *hxk1* mutant strain compared to the WT strain, it is unlikely that Rgt1 is involved in this process, as this protein regulates the expression of multiple genes that are not differentially expressed in the *hxk1* mutant strain (49).

It is interesting that Hxk1 regulates the expression of glucose transporters in different manners. We propose that in the presence of glycerol, Hxk1 favors the expression of *HGT6*, *HGT7*, and *HGT8*, which encode low-affinity glucose transporters, potentially priming the cells for the availability of glucose (51). This ensures efficient glucose uptake, which may enhance the virulence potential of *C. albicans*. The role of Hgt13 in glucose transport is investigated to a lesser extent (37, 51). Previously, it was shown that the *hxk1* mutant strain has an increased glucose transport compared to the WT strain, and this is linked to the increased expression of *HGT13* (21). However, this does not correlate with the transport data of the *HGT13* overexpression strains, indicating that the upregulated expression of *HGT13* in the *hxk1* mutant strain is not caused by the increased amount of glucose transported into these cells. Probably, a gene expression-independent process is responsible for the increased glucose transport into the cells of the *hxk1* mutant strain. For example, it is possible that Hxk1 regulates these transporters by helping them localize to the plasma membrane, thereby regulating their transport capacity.

As observed in the sterol analysis, the amount of zymosterol in the *hxk1* mutant strain cells is lower compared to the WT strain. We assume that this is caused by the upregulated expression of genes with a function downstream of zymosterol (*ERG2*, *ERG3,* and *ERG25*). This can increase the flux toward downstream metabolites of the sterol biosynthesis in the *hxk1* mutant strain. An increased amount of ergosta-5,7-dienol is also found in the *hxk1* mutant strain. This is assumed to be the consequence of the upregulated expression of *ERG3*, which encodes the protein responsible for the conversion of ergosta-7-enol into ergosta-5,7-dienol. As more Erg3 is present, a higher conversion rate can be obtained. The amount of episterol is lower in the *hxk1* mutant strain compared to the WT strain, which is probably due to the increased expression of *ERG3*, which results in a higher flux toward downstream components in the mutant strain compared to the WT strain.

The decreased fluconazole resistance of the *hxk1* mutant strain was not expected, as literature shows that alterations in sterol metabolism mostly result in an increased resistance toward antifungal drugs (52). The reason why Hxk1 influences the sterol metabolism is still unclear. It is known that Hxk1 can play a role in the formation of GPI anchors, which integrate into the cell membrane (15). Although only an effect of Hxk1 is observed in the presence of glycerol, it is possible that under these conditions, Hxk1 plays a role in proper sterol metabolism.

Sod4 and Sod5 are important proteins for the neutralization of ROS surrounding *C. albicans* (27). Since the *hxk1* mutant strain has an upregulation in the expression of these two *SOD* genes, the mutant strain is more tolerant toward H_2_O_2_. This is not linked to increased survival in the presence of macrophages (21). Therefore, we assume that the difference in ROS production by the WT and *hxk1* mutant strains is independent of *SOD* expression. As shown in the literature, the *hxk1* mutant strain is hyperfilamentous when grown in the presence of serum (18, 53). Since the co-culture medium contains serum, the *hxk1* mutant strain is hyperfilamentous when grown in the presence of these macrophages. Therefore, it is possible that macrophages cannot completely engulf the filaments, which can result in frustrated phagosomes (54). These ineffective phagosomes leak damaging radicals, such as ROS molecules, possibly explaining the small peak of ROS molecules produced by the macrophages when infected with the *hxk1* mutant strain.

Different host cell lines have specific characteristics, and *C. albicans* reacts differently toward specific cell types as previously shown in the literature (55). Therefore, one can assume that different features contribute toxicity toward oral and gut epithelial cells. These differential findings observed by others also support our observations when different cell lines were used for toxicity experiments. The increased toxicity of the *hxk1* mutant strain toward gut epithelial cells is not caused by an increased adhesion capacity of this strain. However, we observe a correlation between the deletion of HXK1 and an increased expression of *LIP1* and *PRY1*. Further research is necessary to confirm that the increased toxicity is caused by this increase in gene expression. Lip1 and Pry1 are two important proteins involved in epithelial infections. Lip1 is a lipase that shows lipolytic activity and is part of the *C. albicans* lipase family (45, 56). Lipases are important proteins for *C. albicans* during infection of epithelial cells as they function during active penetration of the epithelial cells (42). Interestingly, *LIP1* seems to be important for gut colonization, as expression levels of *LIP1* were constantly detected when cells infected gastric tissue (57). This indicates the importance of Lip1 during gut epithelial infections and suggests that the increased toxicity of the *hxk1* mutant strain could be caused by an overexpression of *LIP1*. Pry1 is also a secreted, virulence-related protein of *C. albicans*. It is secreted in the presence of lactate, which is also present in the gut (58). Hence, it is assumed that Pry1 is secreted when *C. albicans* cells are present in this host niche and that it plays a role during gut colonization. Therefore, we assume that an increased expression of *PRY1* in the *hxk1* mutant strain in the presence of gut epithelial cells can result in a higher toxicity of the *hxk1* mutant strain toward these gut cells. Furthermore, deletion of *PRY1* results in a decreased adhesion of *C. albicans* cells to host cells (58). It would be interesting to further investigate the effects of the *hxk1* mutant strain on the immune responses of gut epithelial cells (42). Ece1 and Hwp1 are important components in activating the immune responses of epithelial cells upon invading *C. albicans* cells, and both *ECE1* and *HWP1* are upregulated in the *hxk1* mutant strain.

Hxk1 does not bind to the promoters of genes that are differentially regulated in the *hxk1* mutant strain, which confirms an indirect effect of Hxk1 on gene expression. An explanation for our findings may be related to the disruption of the GlcNAc catabolic pathway caused by the deletion of *HXK1*, which would lead to GlcNAc accumulation. Since GlcNAc serves as a signaling molecule, its buildup could activate the GlcNAc signaling pathway. Consequently, it is possible that the gene expression changes we observed in the *hxk1* mutant strain may be due to the activation of the GlcNAc signaling pathway. Therefore, investigating the effect of *HXK1* deletion in very low-density cell cultures would be interesting, as it could help determine whether our observations are based on the function of the Hxk1 protein itself or from the accumulation of GlcNAc.

## MATERIALS AND METHODS

### Growth of *C. albicans* cells

*C. albicans* cells were cultivated in either YP or SC medium, each supplemented with 2% glucose or 3% glycerol, as specified. The YP medium composition consisted of 1% yeast extract (Merck) and 2% bacteriological peptone (BD). The SC medium composition consisted of 0.079% complete CSM (MP Biomedicals), 0.17% yeast nitrogen base without amino acids or ammonium sulfate (Difco), and 0.5% ammonium sulfate (Sigma). Notably, all media could be solidified through the addition of 1.5% agar (Difco).

### Plasmid construction

The CIp10-p*HXK1-mTurq2* plasmid was derived from the CIp10-*mTurq2* plasmid (25). Initially, the CIp10 plasmid was cut with MluI-HF and PstI-HF leading to the removal of the *ACT1* promoter located upstream of the mTurquoise2 gene. Subsequently, the *HXK1* promoter region was amplified from genomic DNA of *C. albicans* and incorporated into the excised plasmid through the utilization of the Gibson assembly kit (NEB). This process was followed by thorough validation of the correct integration of the genetic fragments using PCR analysis.

For the construction of overexpression constructs of *HGT13*, the CIp10 plasmid was used. The plasmid was cut with PstI-HF and XhoI. The *HGT13* gene was amplified from *C. albicans* genomic DNA. Integration of these genes into the cleaved plasmid was facilitated through Gibson assembly. This process was followed by a thorough validation of the correct integration of the genetic fragments, carried out through PCR analysis.

### *C. albicans* strain construction

One microgram of the CIp10-p*HXK1-mTurq2* and CIp10-*HGT13* plasmids was cut with StuI to linearize the plasmids. *C. albicans* cells were grown on YPD medium until early exponential phase. The cells were collected and diluted in lithium acetate (LiAc)/TE buffer. One hundred microliters of cells were added to the linearized plasmid. PEG/LiAc/TE buffer and ssDNA were added to the cells, and the mixture was incubated overnight at 30°C and 300 rpm. The next day, the cells were heat-shocked for 15 minutes at 44°C. Afterwards, the cells were collected, resuspended in YPD medium, and recovered for 4 hours at 30°C. Finally, the cells were plated on YPD nourseothricin (NAT) (200 mg/l; Jena Bioscience) plates to select for colonies that contain the plasmid of interest. Integration of the plasmid in the genome was checked by isolating genomic DNA of the colonies by using phenol/chloroform/isoamyl alcohol (PCI; 50%/48%/2%) extraction, followed by PCR.

The construction of the wild-type C-terminal GFP-tagged Hxk1 strains (Hxk1-GFP) used for CUT&RUN experiments was performed using the CRISPR/Cas9 genome editing protocol described in (59). The resulting strain contains both alleles of *HXK1* tagged with GFP. Briefly, a primer design tool was used to select a gRNA to target the cutting of Cas9 at the 3′ end of *HXK1*. A donor DNA for double-stranded break repair was constructed using a *Candida* clade-optimized eGFP sequence flanked by ~50 bp of homology upstream and downstream of the break site. Using colony PCR, individual colonies were screened to confirm the intended integration of eGFP at the targeted site.

### Copy number determination

The genomic DNA concentration was measured and diluted to 0.5 ng/µL. To define the copy number of the inserted plasmid, a Go-Taq polymerase (Promega) and a StepOnePlus machine (Thermo Fisher) were used. The results were analyzed by using the qBasePlus software (Biogazelle).

### Fluorescence microscopy

The cells were cultured overnight in SC medium supplemented with 3% glycerol. Next, the cells were harvested, washed with sterile MQ water, and diluted to an OD_600_ of 0.1. The cells were then cultivated in SC medium supplemented with 2% glucose until reaching the mid-exponential growth phase. Visual analysis of the cells was performed using a Fluoview FV1000 confocal laser scanning microscope. Specifically, the mTurquoise2 fluorophore was excited utilizing 458 nm light from an argon laser, and emission was captured using a bandpass filter BA 480-495.

### RNA-Seq

RNA-Seq library and data analysis were performed as described in Jenull et al. (2021) with small adaptations (60). Cells were grown on SC glycerol medium at 30°C, and samples were taken before glucose addition and 20 minutes after glucose addition. After collection, the cells were snap-frozen in liquid nitrogen and stored at −80°C. The cells were resuspended in TRIzol Reagent (Thermo Fisher), and 200 µL glass beads (425–600 µm) (Sigma) were added. Afterward, the cells were lysed with a FastPrep instrument (MP Biomedicals) and centrifuged at 14,000 rpm and 4°C for 15 minutes. The supernatants were transferred to an Eppendorf tube, and 200 µL chloroform (ACROS Organics) was added. This solution was centrifuged at 14,000 rpm at 4°C for 15 minutes, supernatants were collected, and 500 µL of isopropanol (VWR) was added. The tubes were placed on ice for 20 minutes to let the RNA precipitate. Next, the RNA was centrifuged, the supernatants were discarded, and the RNA was washed with 70% ice-cold ethanol (Fisher Chemicals). Finally, the RNA was treated with DNase I (New England Biolab). The samples were sent to Novogene (Cambridge, UK) for quality control, library preparation, and sequencing.

Quality controls were performed on the raw data using fastQC v0.11.9, and adapters were removed using cutadapt v3.5 with settings: -interleave -q 30 -O 1. Next, the sequences were aligned against the SC5314 *C. albicans* genome (version SC5314 A22 s07 m01 r140) with NextGenMap V0.5.5 and settings -b -p -q 30. The Picard tools were used to remove the optical read duplicates (settings MarkDuplicates REMOVE_DUPLICATES = true, VALIDATION_STRINGENCY = LENIENT), while the intersect tool from BEDTools was used to remove the mitochondrial reads. Finally, HTseq was used for read counting using the genomic annotation of the SC5314 *C. albicans* genome and settings -f bam -t gene -i ID. Further analysis of differential gene expression was performed in Rstudio using the edgeR package. The false discovery rate (FDR) is the *P* value adjusted for multiple testing using the Benjamini-Hochberg method and was set to a maximum of 0.05. Our RNA-Seq data were submitted to the NCBI GEO database and are publicly accessible under project number PRJNA1074962.

### Gene expression analysis by qPCR

Cells were cultivated in SC medium with 3% glycerol until mid-exponential growth. Next, 50 mM GlcNAc or 2% galactose was added to the cells. After 20 minutes, the cells were collected, washed in ice-cold MQ water, and flash-frozen using liquid nitrogen. The cells were stored at −80°C until further use. For RNA extraction, the cells were dissolved in TRIzol reagent and subsequently subjected to lysis using glass beads and fast prepping. Chloroform, isopropanol, and 70% ethanol were used to collect the RNA from the supernatants. To ensure the removal of DNA components, the extracted RNA was treated with DNase and then converted into cDNA using the iScript cDNA synthesis kit from Bio-Rad. qPCR was conducted using Go-Taq polymerase (Promega) and the StepOnePlus instrument from Thermo Fisher. The ΔΔC_t_ results obtained were subsequently subjected to analysis via the qBasePlus software (Biogazelle) and normalized to WT samples. All primers used for qPCR are listed in Table S1.

### CUT&RUN

For the CUT&RUN experiments, Hxk1-GFP cells were grown on SC 3% glycerol medium overnight at 30°C. After 16 hours, the cells were back-diluted into fresh SC glycerol to an OD_600_ of 0.3. When cells reached an OD_600_ of 1.0, 2% glucose was added to the medium. Cells were collected after 20 minutes by centrifugation at 4,000 × *g* for 5 minutes at room temperature. Cell pellets were snap-frozen in liquid nitrogen and stored at −80°C until ready to isolate nuclei. The CUT&RUN protocol and library preparation were prepared as described in Qasim et al. (2022) (59). Briefly, collected cells were permeabilized to isolate intact nuclei. Anti-GFP antibody (Living Colors Full-Length GFP Polyclonal Antibody; Clontech 632592) was added to the bead-bound nuclei and incubated at 4°C, after which pAG-MNase was added and bound to the target antibody. Upon addition of CaCl_2_, pAG-MNase was activated, and targeted chromatin digestion occurred until the addition of a chelating reagent to inactivate the enzyme. After the enzymatic digestion within the permeabilized nuclei, the pAG-MNase-bound antibody complex and associated DNA fragments diffused out. These fragments were subsequently extracted and purified. Sequencing libraries were prepared from the CUT&RUN-enriched DNA fragments using the NEBNext Ultra II DNA library prep kit (New England Biolabs). The libraries were then run on a 10% PAGE gel to separate and remove any contaminating adapter dimers before sequencing.

The CUT&RUN data from three biological replicates for each sample were analyzed, and binding loci were identified using the workflow published by Qasim et al. (2022) (59). Nonspecific binding was identified in IgG control samples, and these regions were subtracted from the corresponding experimental Hxk1-GFP samples using the subtractBed function in bedtools (version 2.30.0) using the default workflow parameters (59). In addition, IgG-subtracted binding loci overlapping highly repetitive regions, telomeric repeats, and centromeres were excluded to reduce false-positive results. IgG-subtracted peaks present in all three of the replicates were selected to identify genes bound by Hxk1. Concordance between replicates was evaluated using the ChIPpeakAnno package (version 3.28.1) in R (version 4.1.2) (61). The binding loci in the three biological replicates were annotated to the nearest gene in both the forward and reverse strands in the *C. albicans* genome using a custom Python script. Genes were annotated as bound by Hxk1 if the Hxk1 binding peak was identified in the upstream region of the corresponding gene in the three biological replicates. The upstream region of a gene is defined as the 5′-flanking region spanning the start codon of the putative target gene and the stop codon of its immediate upstream gene in the same strand. Binding peaks were assigned to a gene if they overlap their corresponding 5′-flanking region irrespective of whether they overlap an open reading frame in the opposite strand. Our CUT&RUN data were submitted to the NCBI GEO database and are publicly accessible under accession number GSE254730.

### Glucose transport assays

The glucose transport assays were performed according to a previously described method (21). Cells were cultured overnight in SC glycerol (3%) at 30°C. The cells were harvested via centrifugation and washed twice with MES buffer (10 mM; pH 7). Afterward, the cells were resuspended in SC medium to reach a final concentration of 125 mg/mL. At the start of the experiment, the cells were incubated at 30°C for 10 minutes. Following this, a solution of 100 mM Tris-MES buffer (pH 5.5) was added to the cells, along with a radioactive 0.05% ^14^C-glucose solution (1,500 cpm/nmol glucose; PerkinElmer). After 10 seconds of incubation, the uptake of glucose was halted through the addition of ice-cold MQ water. The cells were subsequently gathered on a filter, and this filter was put in scintillation fluid (Lumagel Safe; PerkinElmer). A scintillation counter (Hidex) was used to quantify the amount of radioactivity on the filter.

### Ergosterol measurements

Sterols were extracted and analyzed based on Morio et al. with some modifications (62). Stationary-phase cultures were obtained by growing cells in SC medium with 3% glycerol in a shaking incubator at 37°C for 48 hours. Cells were harvested by centrifugation, washed twice with MQ water, and a pellet of 20 mg of cells was stored at −80°C. The pellet was resuspended (vortexing for 1 minute) in 300 µL saponification medium (12.5 g KOH [Sigma] in 18 mL MQ water diluted to 50 mL with 98% ethanol), transferred to a 2 mL capped glass vial, and incubated at 80°C for 1 hour in a shaking water bath. Sterols were extracted by adding 100 µL MQ water and 400 µL hexane (VWR), including 1 µL of 5 mg/mL 5-a-cholestane as an internal standard (Sigma), followed by vortexing for 3 minutes, 20 minutes of phase separation, and collection of 350 µL of the top (hexane) layer. A second extraction was done by adding 600 µL hexane, followed by vortexing for 3 minutes, 20 minutes of phase separation, and collection of 550 µL of the top (hexane) layer. The two collected hexane fractions were combined and dried using vacuum centrifugation (Automatic Environmental SpeedVac System AES2010) for 30 minutes at room temperature. Sterol extracts were re-dissolved in 60 µL hexane and derivatized by adding 10 µL of a silylating mixture (Sigma), followed by short vortexing and incubation at room temperature for at least 1 hour. Derivatized extracts were shortly centrifuged to precipitate potential debris, and 50 µL of the extract was transferred to a smaller insert glass tube for GC-MS analysis.

The samples were analyzed using a Thermo Scientific GC-MS (Trace 1300 - ISQ QD) equipped with a TriPlus RSH autosampler and a Restek Rxi-5ms capillary GC column (30 m × 0.25 mm ID). Helium was used as a carrier gas with a flow rate of 1.4 mL/min. Injection was carried out at 250°C in split mode after 1 minute and with a ratio of 1:10. The temperature was first held at 50°C for 1 minute and then allowed to rise to 260°C at a rate of 50°C/min, followed by a second ramp of 2°C/min until 325°C was reached; that temperature was maintained for 3 minutes. The mass detector was operated in scan mode (50 to 600 atomic mass units), using electron impact ionization (70 eV). The temperatures of the MS transfer line and detector were 325°C and 250°C, respectively. Sterols were identified by their retention time relative to the internal standard (cholestane) and specific mass spectrometric patterns using Chromeleon software (version 7). The spectra were matched to GC-MS libraries described in 63 and NIST/EPA/NIH version 2. Analysis was performed by integration over the base ion of each sterol. Abundance was calculated relative to the internal standard by comparing the relative peak areas of the compounds. Sterol extraction and analysis of each strain was performed in triplicate (technical repeats) on individually cultured strains.

### Broth dilution assays

The broth dilution assays were based on previously described methods (64). *C. albicans* cells were cultured overnight in YP glycerol medium at 30°C. The cells were collected, washed three times with 1× PBS, and diluted to 4 × 10^3^ cells/mL in RMPI medium with 3% glycerol. *C. albicans* cells were incubated at 37°C with a one-half fluconazole dilution series between 0 and 32 µg/mL. After 48 hours, the OD_600_ was measured, and a dose-response curve was plotted. The MIC values were calculated by normalization to the OD_600_ of the cells without fluconazole. The MIC50 is the drug concentration at which 50% of growth was inhibited compared to the no-drug OD_600_.

### *In vitro* H_2_O_2_ stress assay

*C. albicans* cells were cultured overnight in YP medium supplemented with 2% glucose. Next, the cells were harvested through centrifugation and washed twice with sterile MQ water. The samples were diluted to achieve an OD_600_ of 0.1, and a dilution series was prepared by sequentially diluting (three times) the samples in a 1:10 ratio. Subsequently, 5 µL of each dilution was spotted on SC glucose (2%) plates supplemented with 2 mM H_2_O_2_. The plates were incubated at 30°C with images captured after 24 hours.

### Cultivation of primary bone marrow-derived macrophages

Bone marrow was obtained from the femur and tibia of C57BL/6J mice in accordance with previously established methods (65). The extracted cells were subsequently subjected to differentiation to obtain bone marrow-derived macrophages (BMDMs). This differentiation process involved cultivating the cells within differentiation medium (Dulbecco’s modified Eagle’s medium [DMEM; Gibco], 10% heat-inactivated fetal calf serum (FCS) (Sigma), 100 U/mL penicillin, 100 µg/mL streptomycin, and L-conditioned media).

### Macrophage ROS accumulation assay

BMDMs were seeded in a Nunc F96 MicroWell White Polystyrene Plate (5 × 10^4^ cells/well) in differentiation medium. These BMDMs were then subjected to infection with *C. albicans* cells at a concentration of 2.5 × 10^5^ cells/well. Fifty micrograms of a luminol solution, containing HBSS (Gibco), luminol sodium salt (Sigma), and horseradish peroxidase (HRP) from horseradish type IV (Sigma), were added to the cells. The luminescence produced by the macrophages was recorded over a span of 2 hours utilizing the Synergy microplate reader (BioTek).

### qPCR of *C. albicans* cells after cultivation with macrophages

The qPCR experiment of *C. albicans* cells co-cultured with macrophages was performed according to Tucey et al. (2018) (66). Macrophages were seeded in a 6-well plate at a density of 1.6 × 10^6^ cells/well. The next day, *C. albicans* cells were added to the macrophages at a MOI of 10. Three hours post-infection, 4% Triton-PBS was added to the co-culture to lyse the mammalian cells. The fungal cells were collected, and RNA was isolated as previously stated.

### Cultivation of mammalian cell lines

TR146 cells (ECACC General collection) were cultivated in DMEM/Nutrient Mixture F-12 medium (Gibco) supplemented with 10% heat-inactivated FCS (Gibco) and 50,000 U penicillin-streptomycin (Gibco) at 37°C and 5% CO_2_. Similarly, CaCo2 cells (ATCC) were cultivated in MEM medium (Gibco) supplemented with 10% heat-inactivated FCS (Gibco) and 50,000 U penicillin-streptomycin (Gibco) at 37°C and 5% CO_2_. Regular sub-culturing of cells was performed every two to three days to ensure their continued growth and viability.

### Toxicity toward epithelial cells

Mammalian cells were seeded in Nunc F96 MicroWell plates (Thermo Fisher) at a density of 1 × 10^4^ cells/well and incubated for 24 hours at 37°C and 5% CO_2_. *C. albicans* cells were collected and washed with 1× PBS and diluted to a concentration of 1 × 10^7^ cells/mL. These cells were then co-cultured with the mammalian cells for a duration of 24 hours. Post-incubation, the CyQUANTUM LDH Cytotoxicity Assay Kit (Invitrogen) was used to quantify the lactate dehydrogenase (LDH) release by the mammalian cells. Specifically, 50 µL of the supernatant was collected and incubated with 50 µL of reaction buffer in the dark for 30 minutes at room temperature. Absorbance measurements at 490 nm and 680 nm were subsequently conducted using the Synergy microplate reader (BioTek).

### Adhesion toward epithelial cells

Mammalian cells were seeded in Nunc F96 MicroWell plates (Thermo Fisher) at a density of 2 × 10^4^ cells/well and subsequently incubated for three days at 37°C and 5% CO_2_. *C. albicans* cells were harvested and washed with 1× PBS. Afterwards, the cells were diluted to a concentration of 1 × 10^5^ cells/mL and added to the mammalian cells. After an incubation period of 90 minutes, the wells were washed to remove any non-adherent *C. albicans* cells. The adhered cells were treated with 1× trypsin for 10 minutes at 37°C. A 10% FCS-PBS solution was added to the wells. The cells were collected and plated for colony-forming units (CFU).

### qPCR of *C. albicans* cells after cultivation with epithelial cells

Epithelial cells were seeded in a 6-well plate at a density of 1.6 × 10^5^ cells/well and incubated for 48 hours at 37°C and 5% CO_2_. Afterwards, the epithelial cells were infected with *C. albicans* cells at MOI 20. Six hours post-infection, 4% triton-PBS was added to the co-culture to lyse the mammalian cells. The fungal cells were collected, and RNA was isolated as previously stated.

### Statistical analysis

Statistical analysis was done using GraphPad Prism. The RNA-Seq data were analyzed with R Studio.

## Data Availability

Both RNA-seq and CUT&RUN datasets are publicly available. RNA-seq datasets are available at the NCBI Sequence Read Archive (SRA) with accession numbers SRX23597271, SRX23597286, SRX23597272, SRX23597273, SRX23597279, SRX23597274, SRX23597280, and SRX23597275. The CUT&RUN dataset is available on Gene Expression Omnibus (GEO) with accession number GSE254730.

## SUPPLEMENTAL MATERIAL

Data set S1 msphere.00395-25-s0001.xls Genes differentially expressed before glucose addition.

Data set S2 msphere.00395-25-s0002.xls Genes differentially expressed after glucose addition.

Data set S3 msphere.00395-25-s0003.xlsx Locations where Hxk1 is bound to the genome.

Data set S4 msphere.00395-25-s0004.pdf Binding peaks in the upstream region of 28 genes called as bound by Hxk1.

Figure S1 msphere.00395-25-s0005.tif The expression of HGT13 was measured in an HGT13 overexpression strain.

Table S1 msphere.00395-25-s0006.docx qPCR primers used in this study.

ASM does not own the copyrights to Supplemental Material that may be linked to, or accessed through, an article. The authors have granted ASM a non-exclusive, world-wide license to publish the Supplemental Material files. Please contact the corresponding author directly for reuse.
