# Supplementary material for: Candida albicans Hxk1 influences expression of metabolic- and virulence-related genes
Source: mSphere. 2025 Sep 25;10(10):e00395-25. doi: 10.1128/msphere.00395-25 (PMC12570500; doi:10.1128/msphere.00395-25)
Supplement: Data set S4 — Binding peaks in the upstream region of 28 genes called as bound by Hxk1. [file msphere.00395-25-s0004.pdf]

Dataset S4

Binding peaks in the upstream intergenic regions of the 28 genes called as bound by Hxk1 are shown. Peak enrichment is shown in the top panel along with scaled distance of the x-axis. Genomic coverage of Hxk1-GFP binding is averaged across biological replicates and is shown in blue. Genomic coverage of IgG control binding is averaged across biological replicates and is shown in red. Genomic coverage is scaled between the experimental and control samples across each plot. The bottom panel shows ORFs as grey boxes with arrows indicating directionality. The blue boxes are the called significant peak regions based on subtracting the IgG control signal from the Hxk1-GFP signal for each biological replicate.

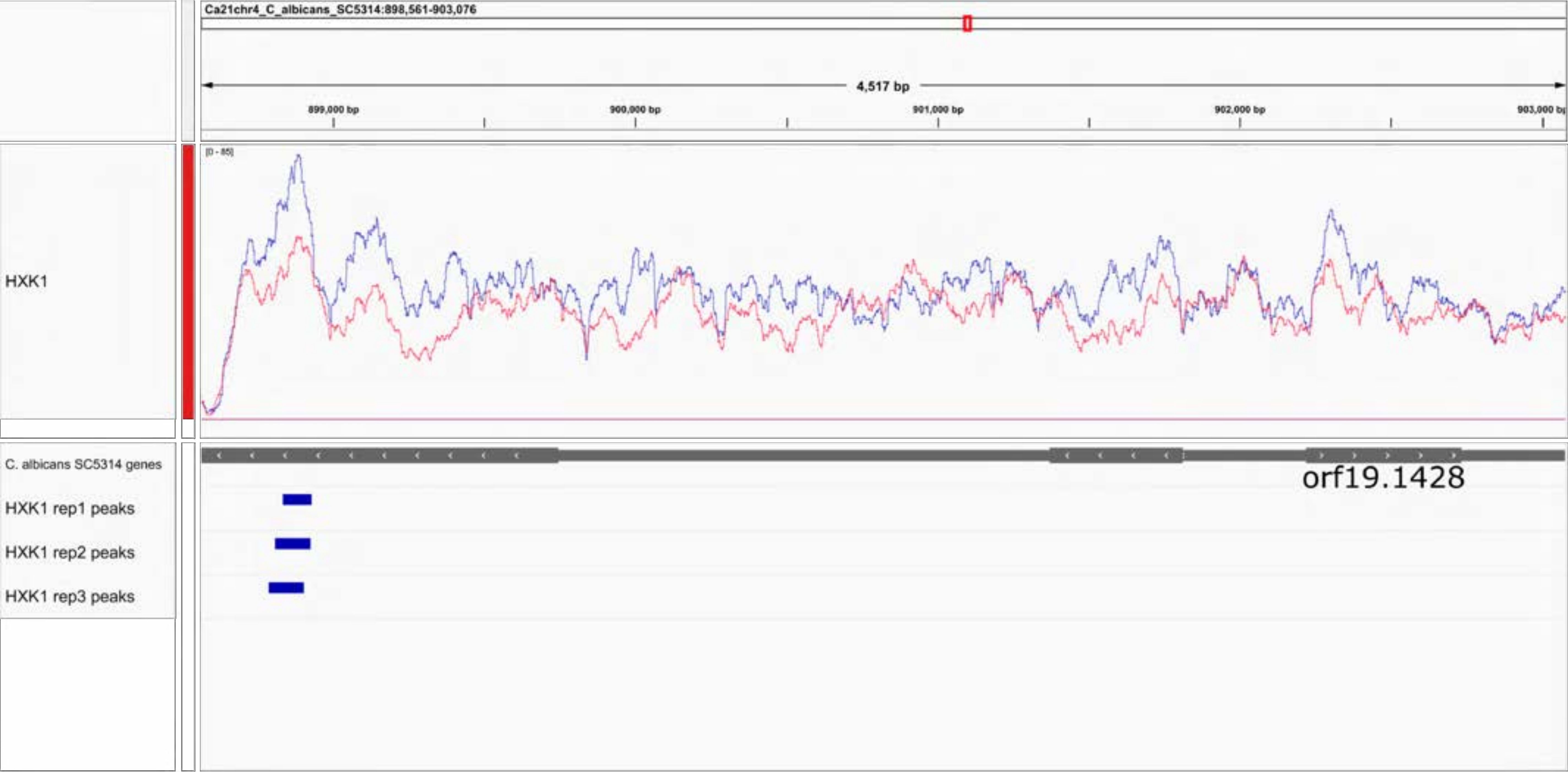

Ca21chr4_C_albicans_SC5314:898,561-903,076
4,517 bp
899,000 bp
900,000 bp
901,000 bp
902,000 bp
903,000 bp
HXK1
[0 - 85]
C. albicans SC5314 genes
orf19.1428
HXK1 rep1 peaks
HXK1 rep2 peaks
HXK1 rep3 peaks

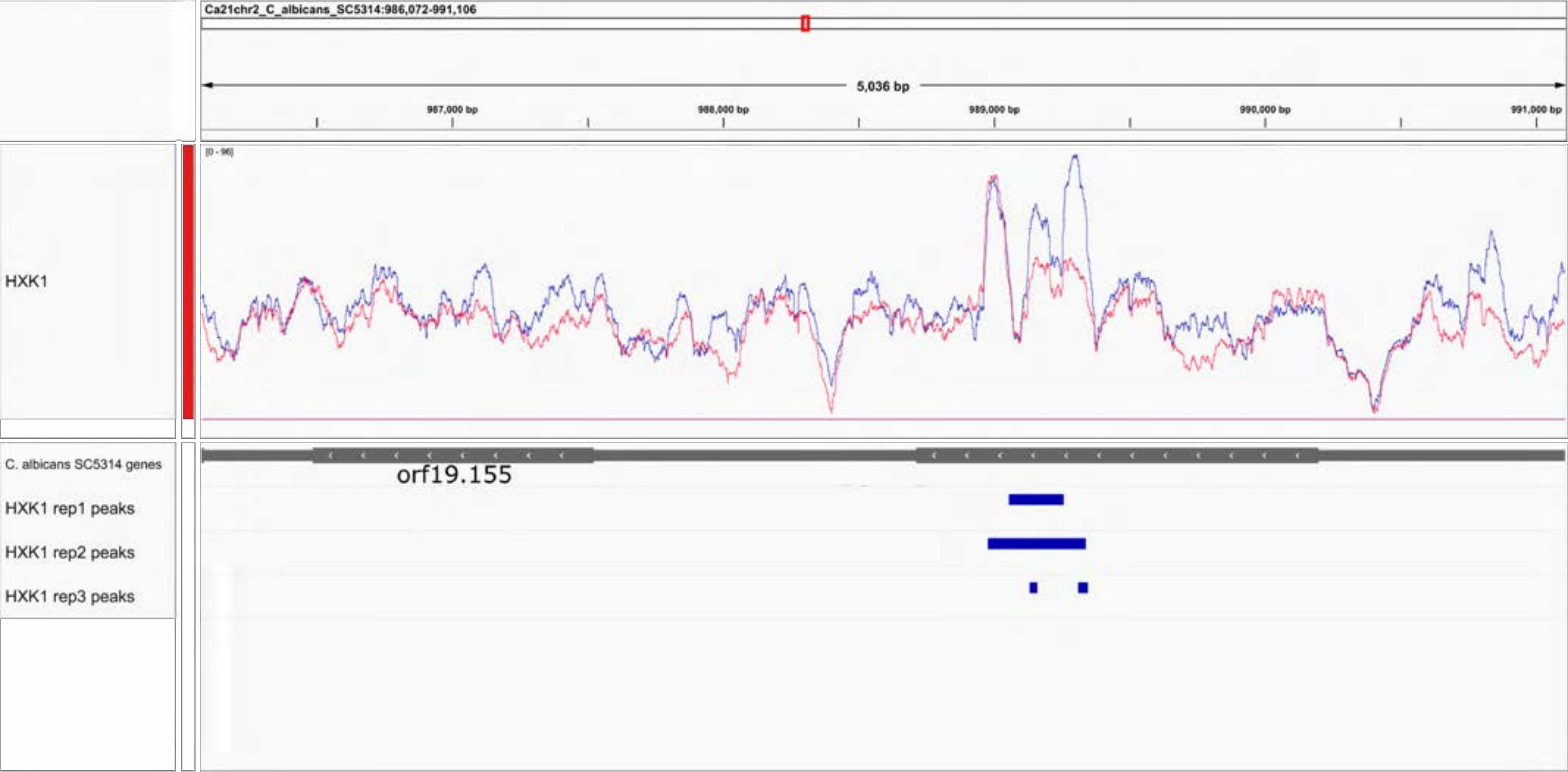

Ca21chr2_C_albicans_SC5314:986,072-991,106
5,036 bp
987,000 bp
988,000 bp
989,000 bp
990,000 bp
991,000 bp
HXK1
[0 - 96]
C. albicans SC5314 genes
orf19.155
HXK1 rep1 peaks
HXK1 rep2 peaks
HXK1 rep3 peaks

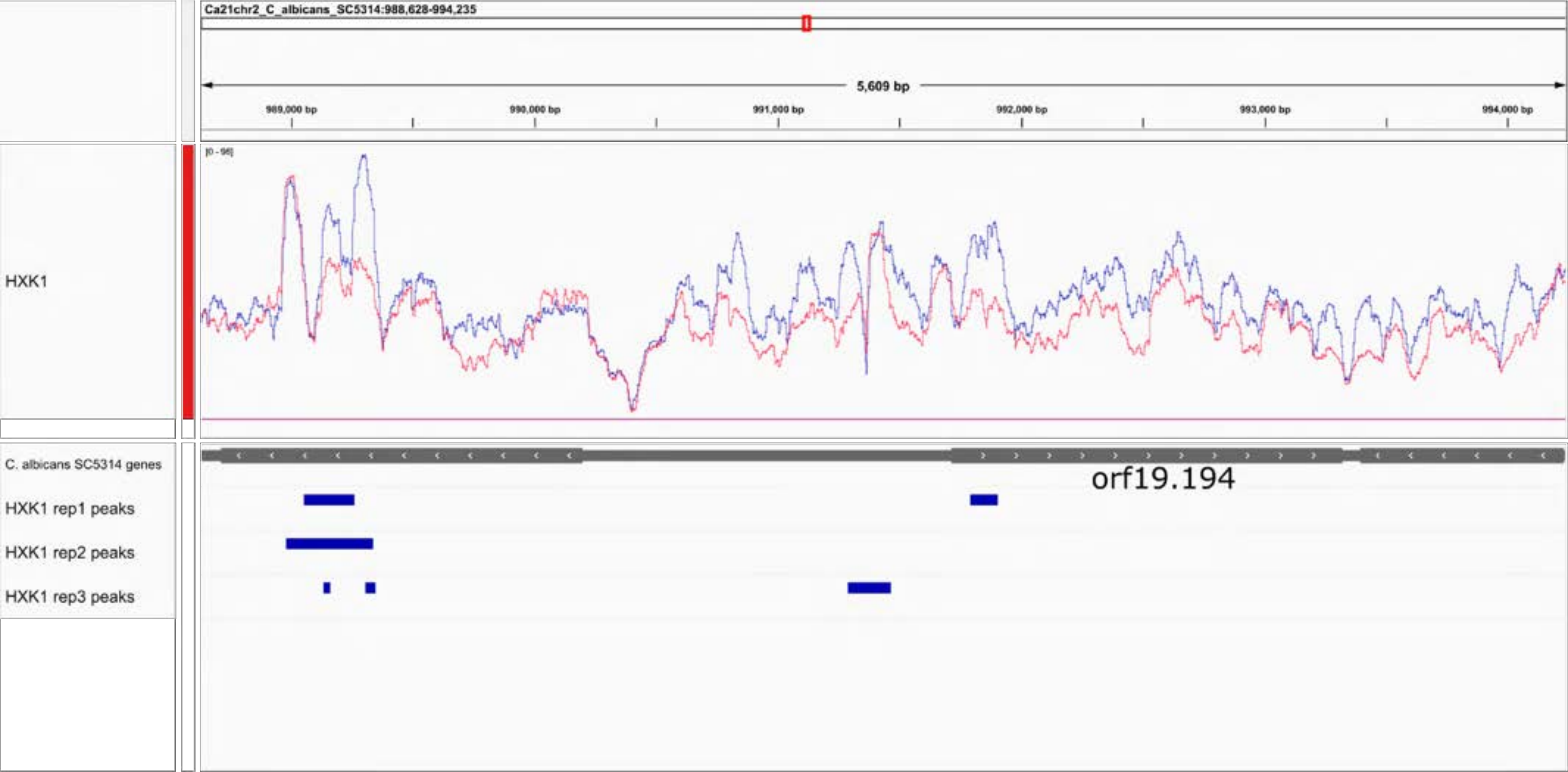
Ca21chr2_C_albicans_SC5314:988,628-994,235
5,609 bp
989,000 bp
990,000 bp
991,000 bp
992,000 bp
993,000 bp
994,000 bp
HXK1
[0 - 96]
C. albicans SC5314 genes
orf19.194
HXK1 rep1 peaks
HXK1 rep2 peaks
HXK1 rep3 peaks

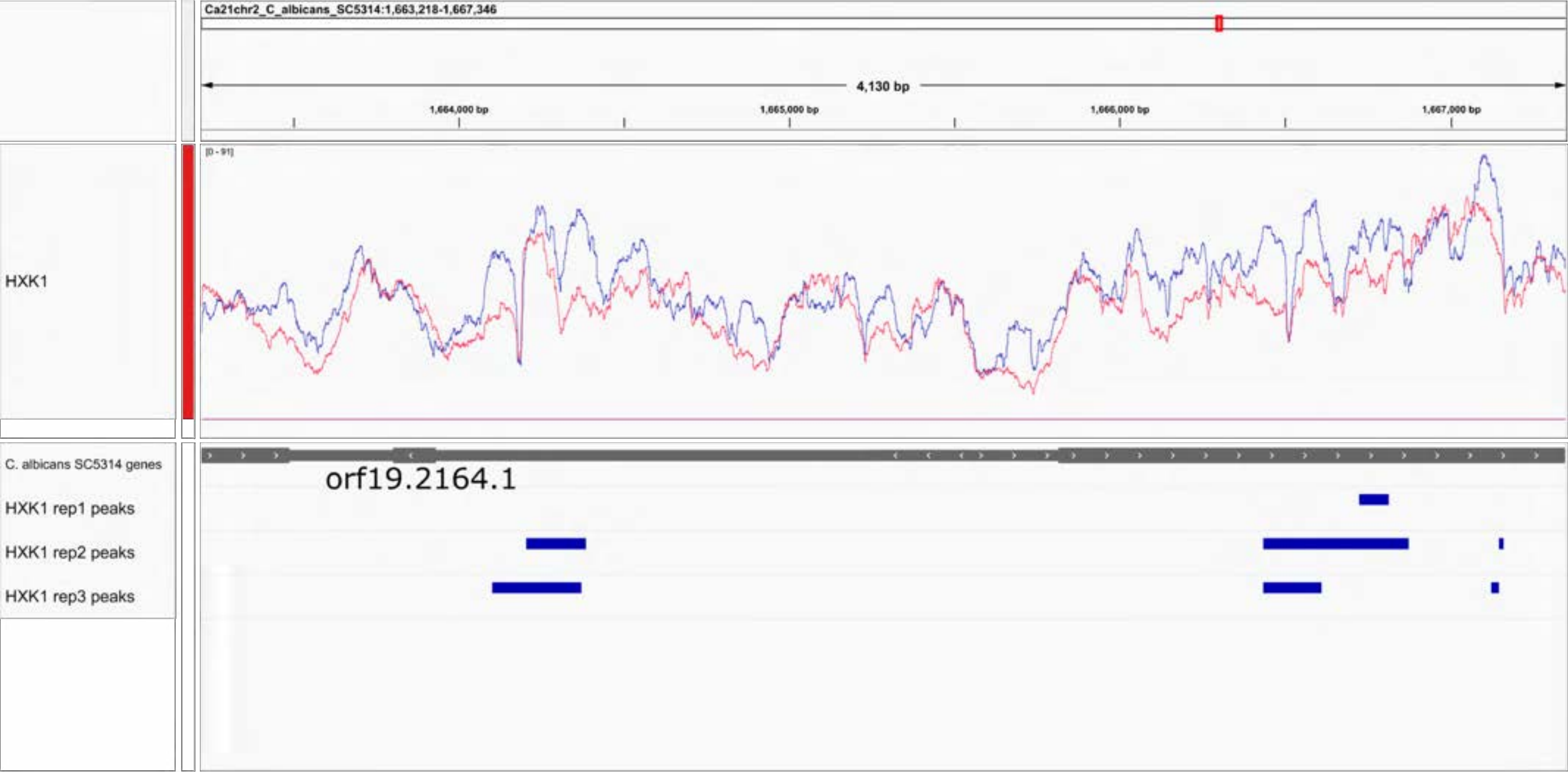
Ca21chr2_C_albicans_SC5314:1,663,218-1,667,346
4,130 bp
1,664,000 bp
1,665,000 bp
1,666,000 bp
1,667,000 bp
HXK1
[0 - 91]
C. albicans SC5314 genes
orf19.2164.1
HXK1 rep1 peaks
HXK1 rep2 peaks
HXK1 rep3 peaks

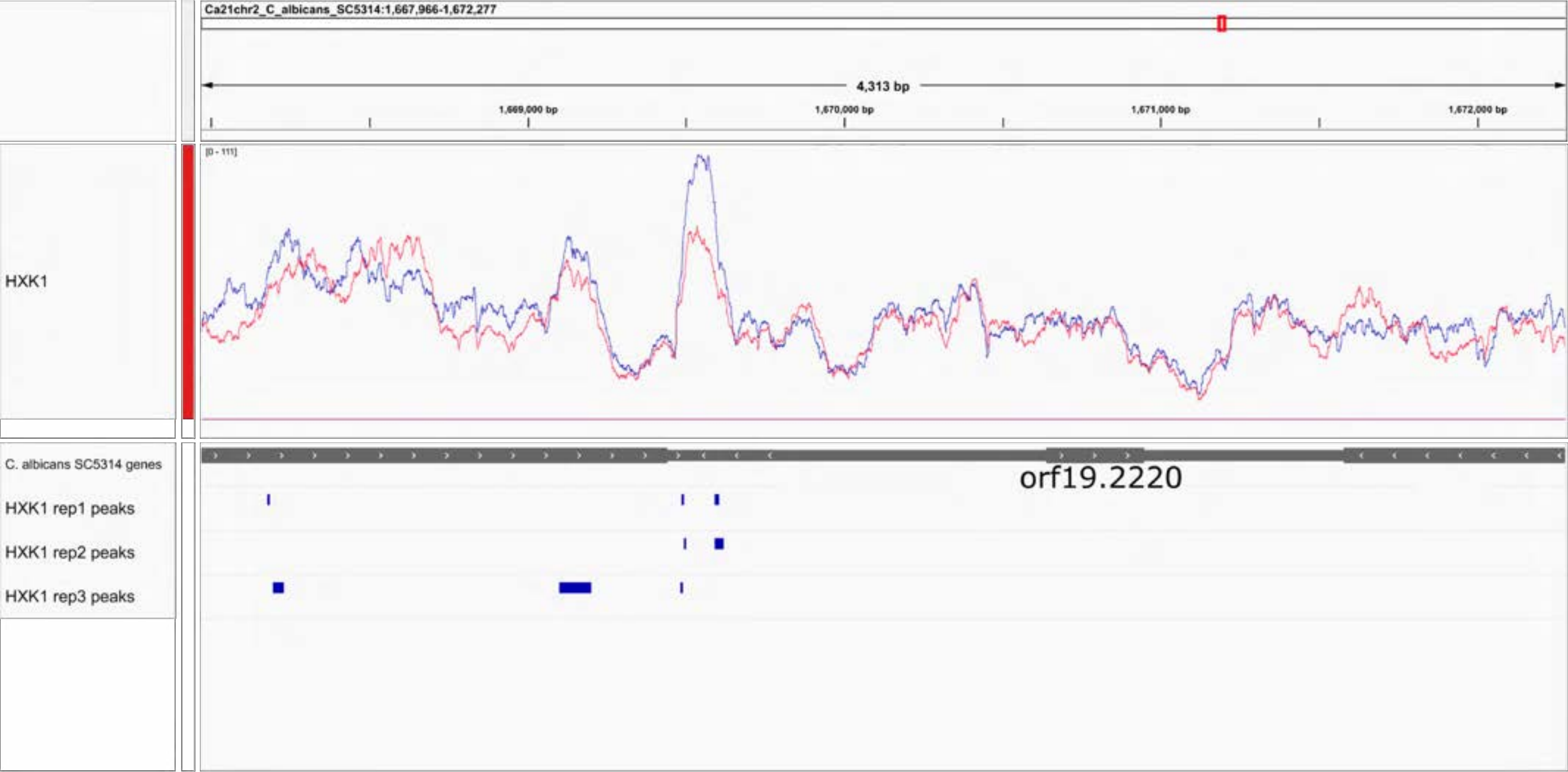

Ca21chr2_C_albicans_SC5314:1,667,966-1,672,277
4,313 bp
1,669,000 bp
1,670,000 bp
1,671,000 bp
1,672,000 bp
HXK1
[0 - 111]
C. albicans SC5314 genes
orf19.2220
HXK1 rep1 peaks
HXK1 rep2 peaks
HXK1 rep3 peaks

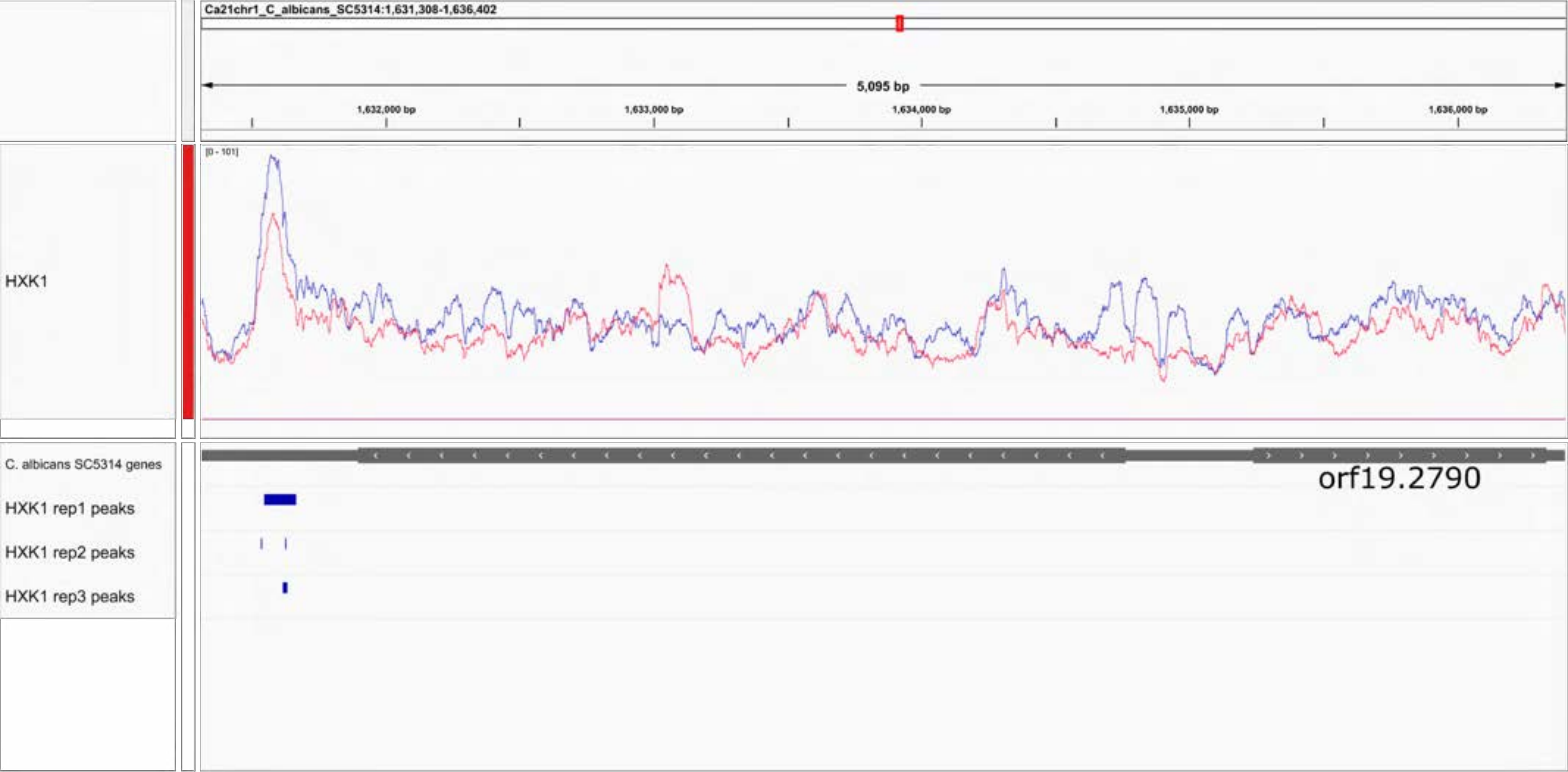
Ca21chr1_C_albicans_SC5314:1,631,308-1,636,402
5,095 bp
1,632,000 bp
1,633,000 bp
1,634,000 bp
1,635,000 bp
1,636,000 bp
[0 - 101]
HXK1
C. albicans SC5314 genes
HXK1 rep1 peaks
HXK1 rep2 peaks
HXK1 rep3 peaks
orf19.2790

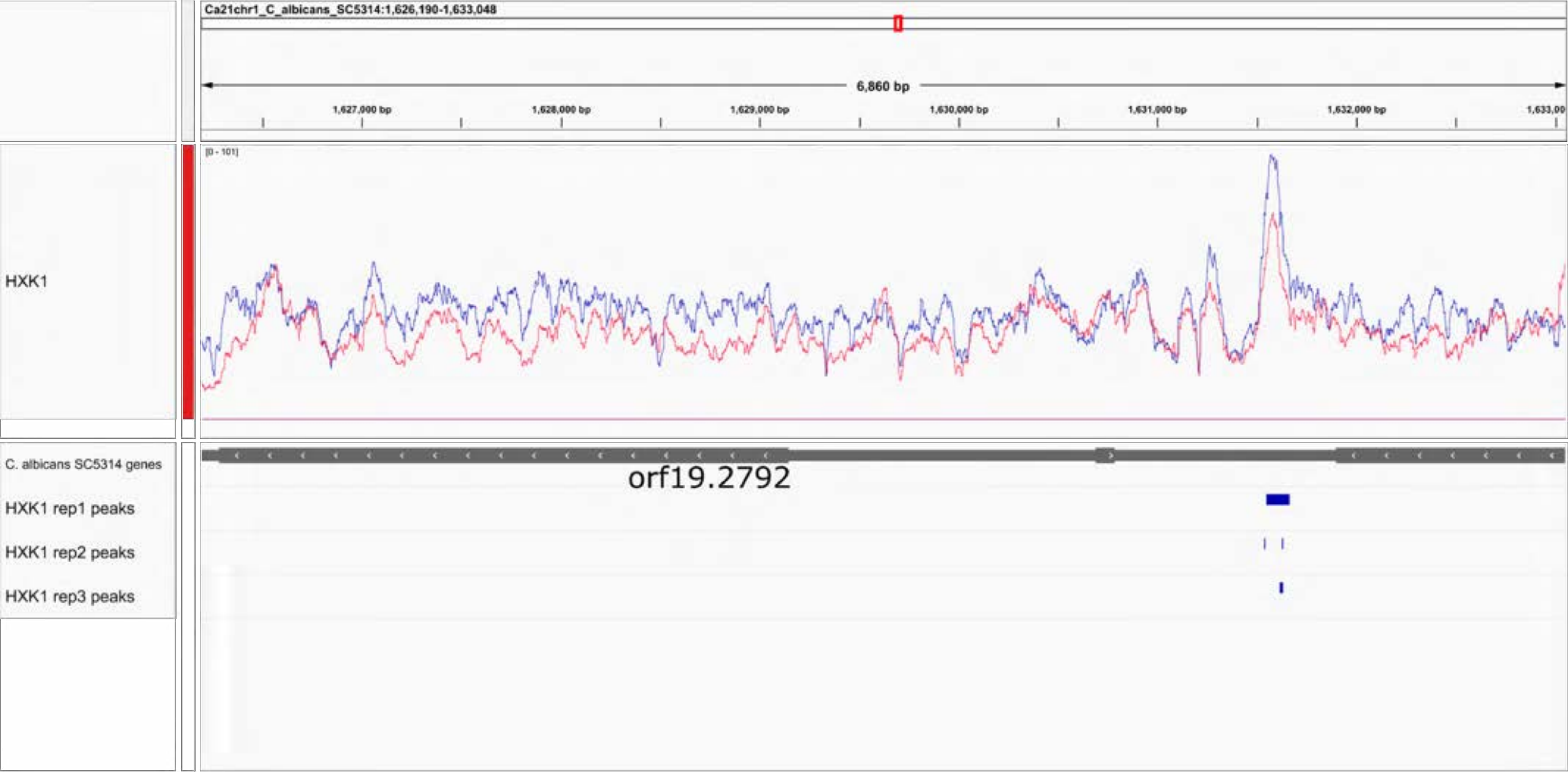
Ca21chr1_C_albicans_SC5314:1,626,190-1,633,048
6,860 bp
1,627,000 bp
1,628,000 bp
1,629,000 bp
1,630,000 bp
1,631,000 bp
1,632,000 bp
[0 - 101]
HXK1
C. albicans SC5314 genes
orf19.2792
HXK1 rep1 peaks
HXK1 rep2 peaks
HXK1 rep3 peaks

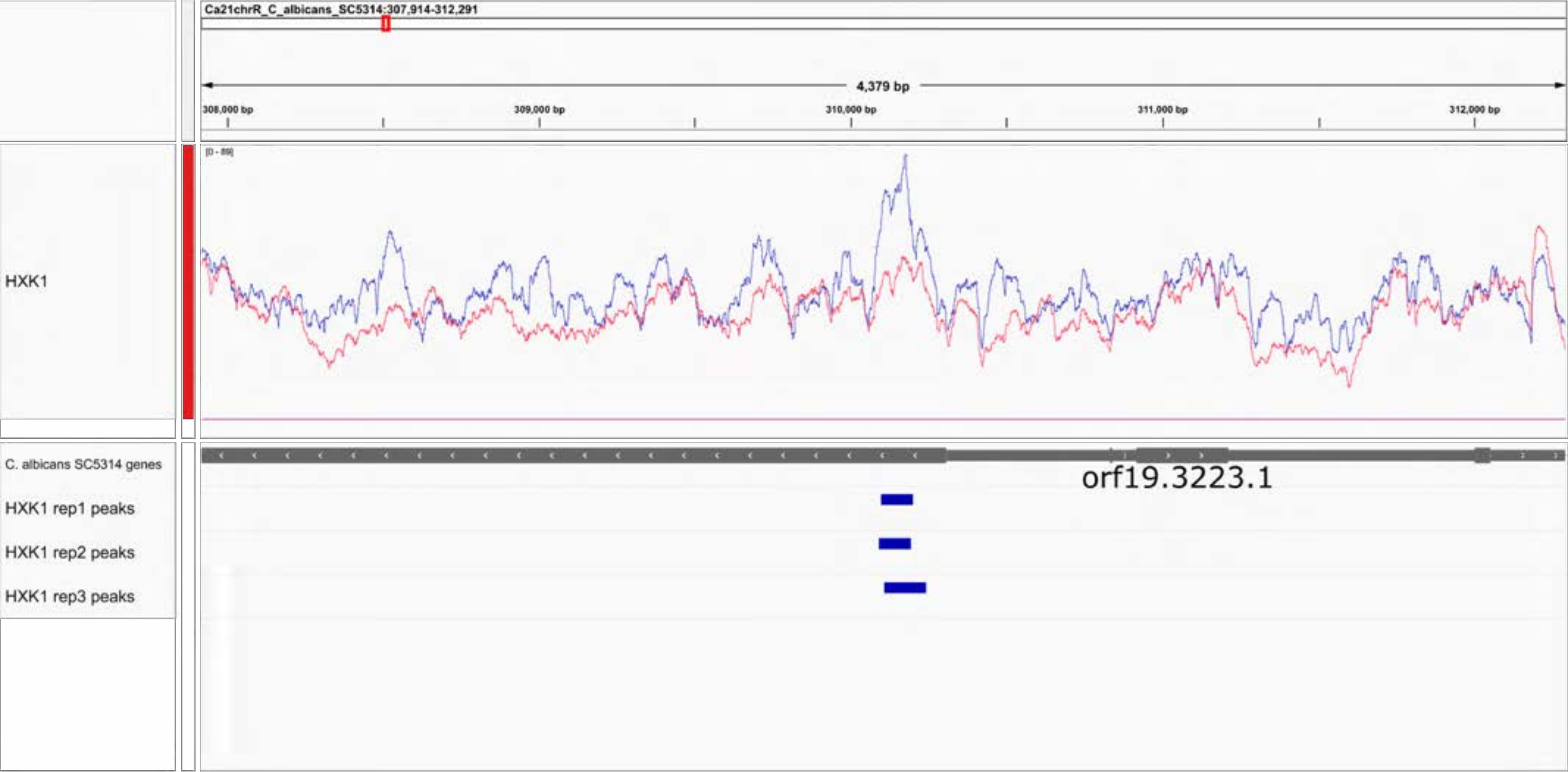
Ca21chrR_C_albicans_SC5314:307,914-312,291
4,379 bp
308,000 bp
309,000 bp
310,000 bp
311,000 bp
312,000 bp
HXK1
C. albicans SC5314 genes
orf19.3223.1
HXK1 rep1 peaks
HXK1 rep2 peaks
HXK1 rep3 peaks

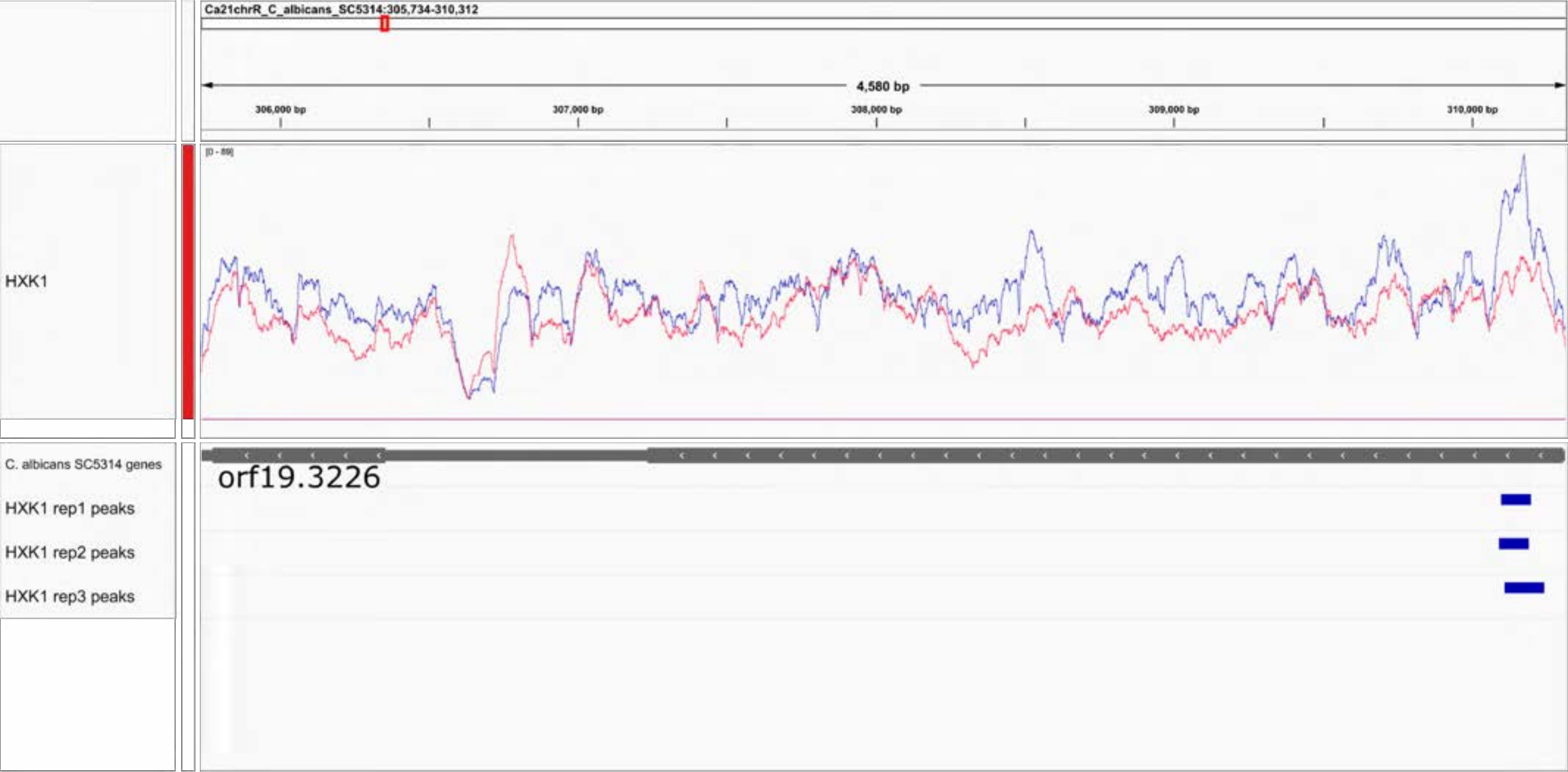
Ca21chrR_C_albicans_SC5314:305,734-310,312
4,580 bp
306,000 bp
307,000 bp
308,000 bp
309,000 bp
310,000 bp
HXK1
C. albicans SC5314 genes
orf19.3226
HXK1 rep1 peaks
HXK1 rep2 peaks
HXK1 rep3 peaks

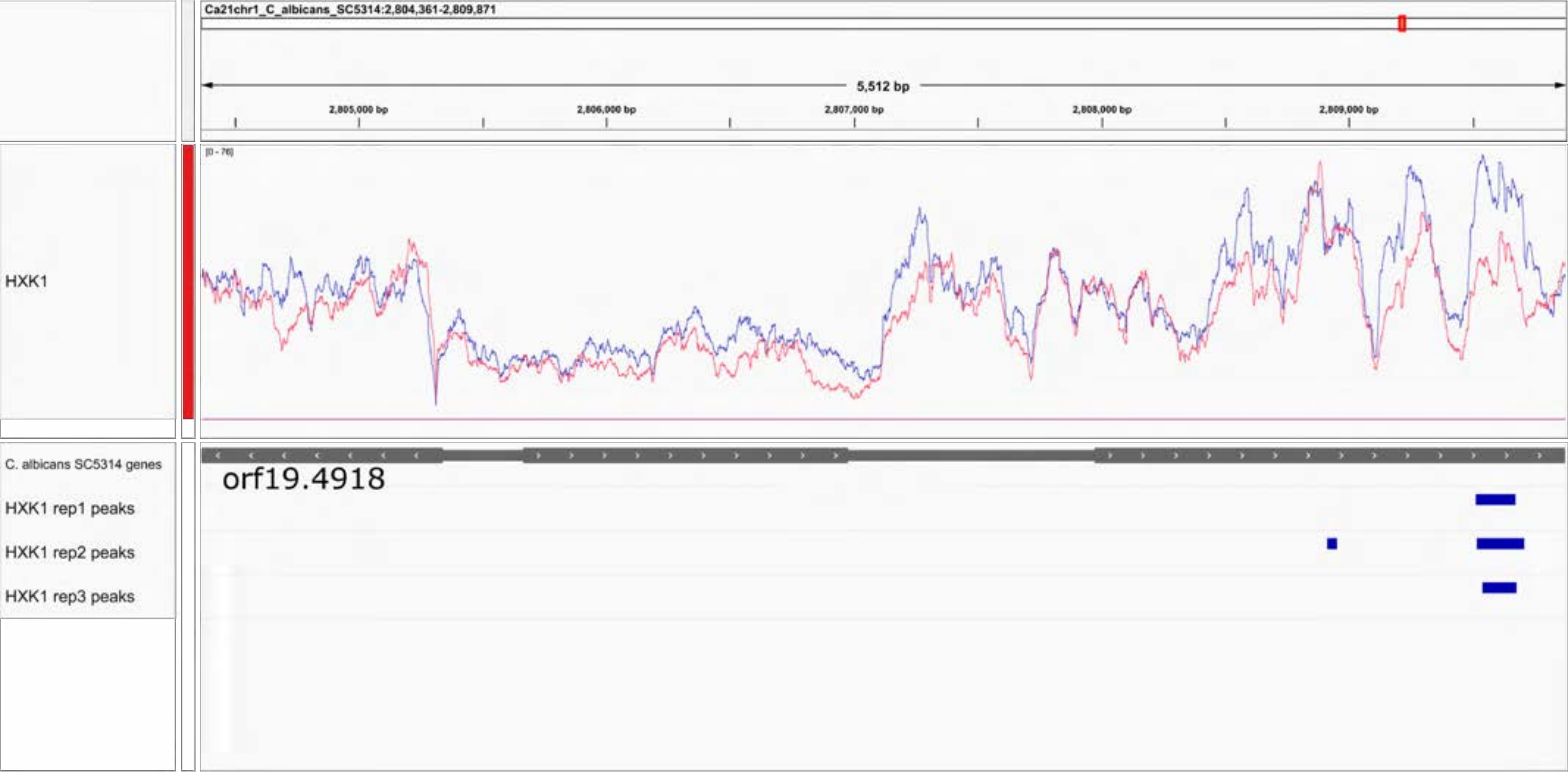

Ca21chr1_C_albicans_SC5314:2,804,361-2,809,871
5,512 bp
2,805,000 bp
2,806,000 bp
2,807,000 bp
2,808,000 bp
2,809,000 bp
[0 - 76]
HXK1
C. albicans SC5314 genes
orf19.4918
HXK1 rep1 peaks
HXK1 rep2 peaks
HXK1 rep3 peaks

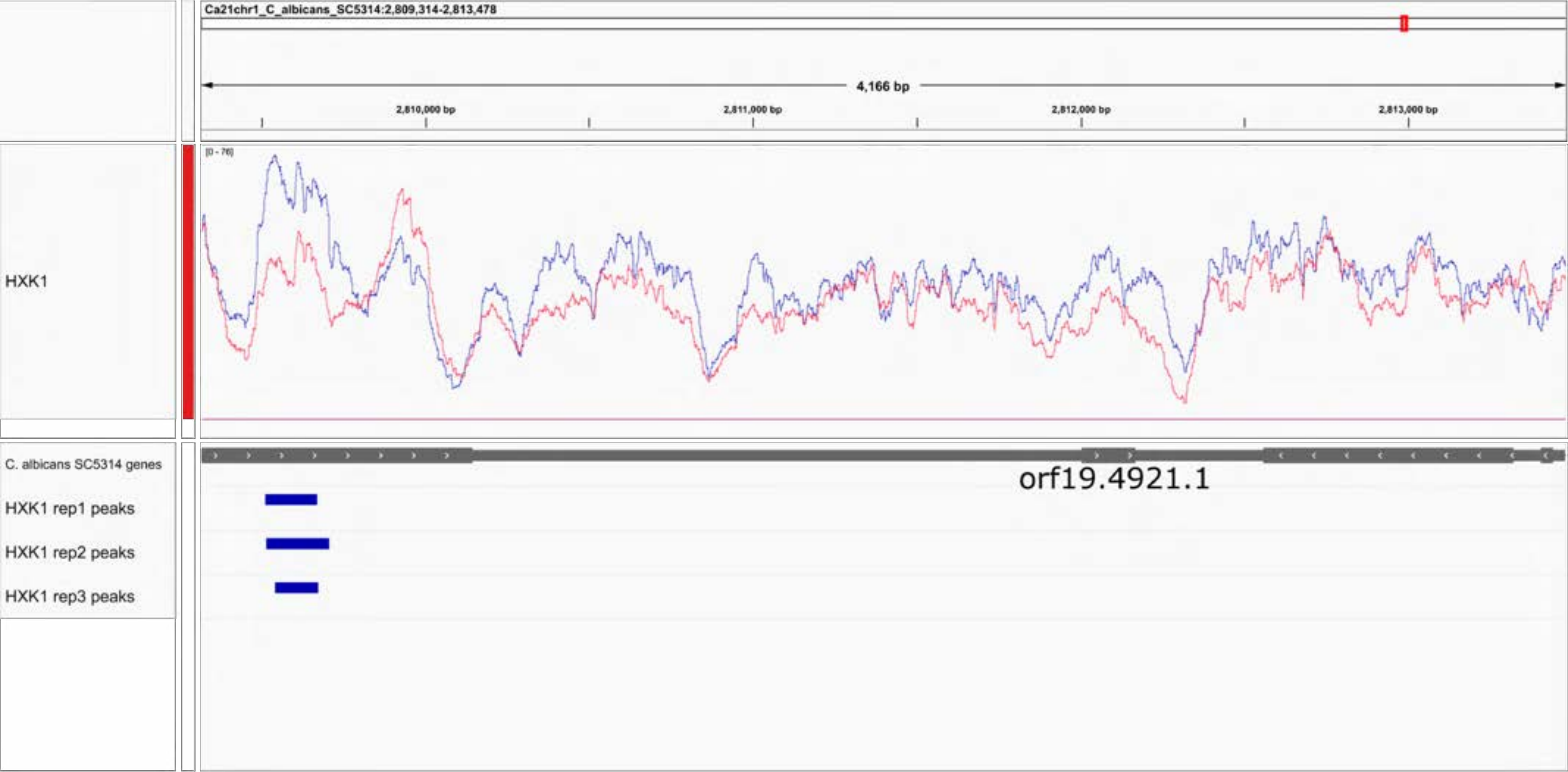

Ca21chr1_C_albicans_SC5314:2,809,314-2,813,478
4,166 bp
2,810,000 bp
2,811,000 bp
2,812,000 bp
2,813,000 bp
HXK1
C. albicans SC5314 genes
orf19.4921.1
HXK1 rep1 peaks
HXK1 rep2 peaks
HXK1 rep3 peaks

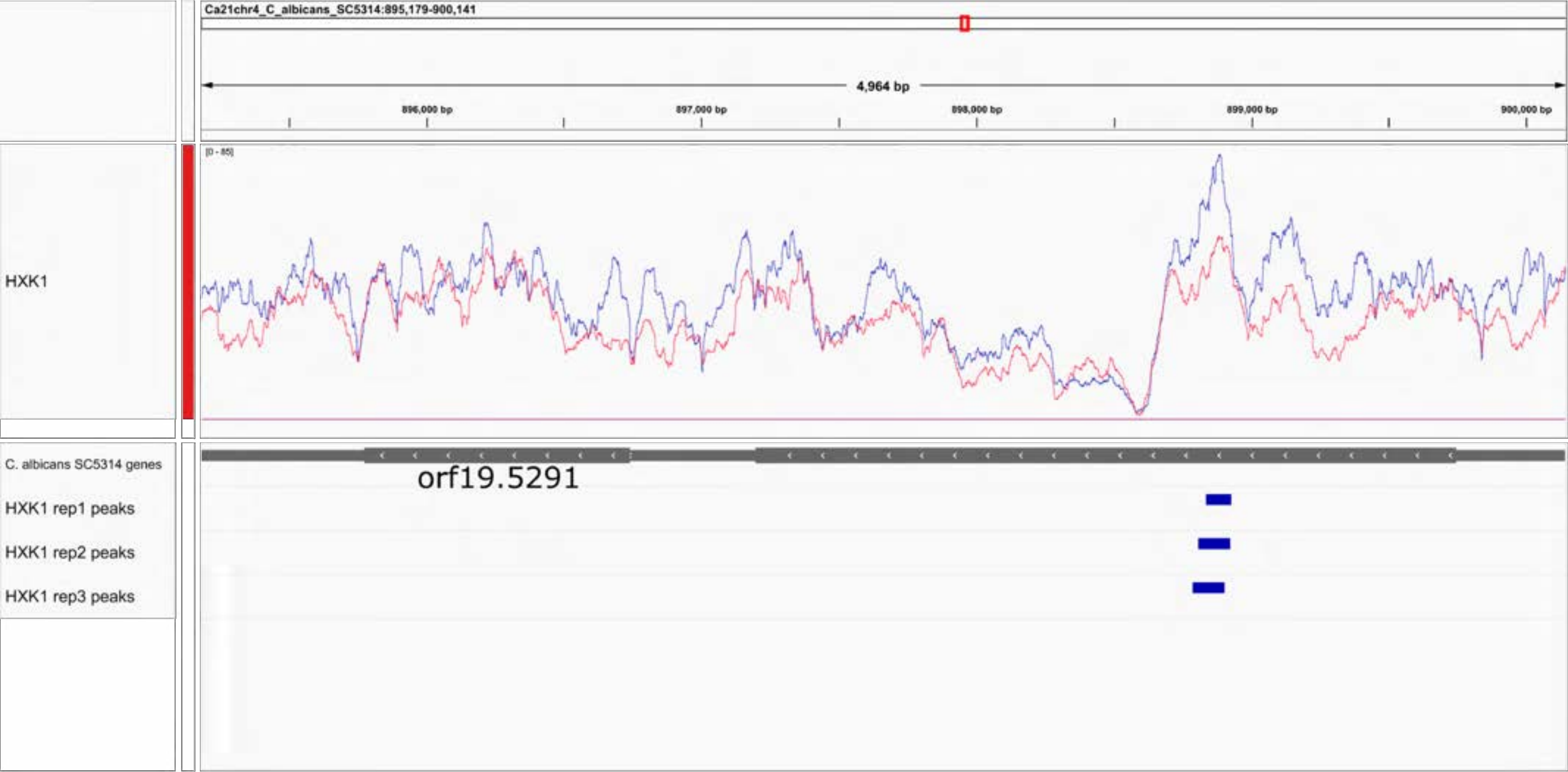

Ca21chr4_C_albicans_SC5314:895,179-900,141
4,964 bp
896,000 bp
897,000 bp
898,000 bp
899,000 bp
900,000 bp
HXK1
C. albicans SC5314 genes
orf19.5291
HXK1 rep1 peaks
HXK1 rep2 peaks
HXK1 rep3 peaks

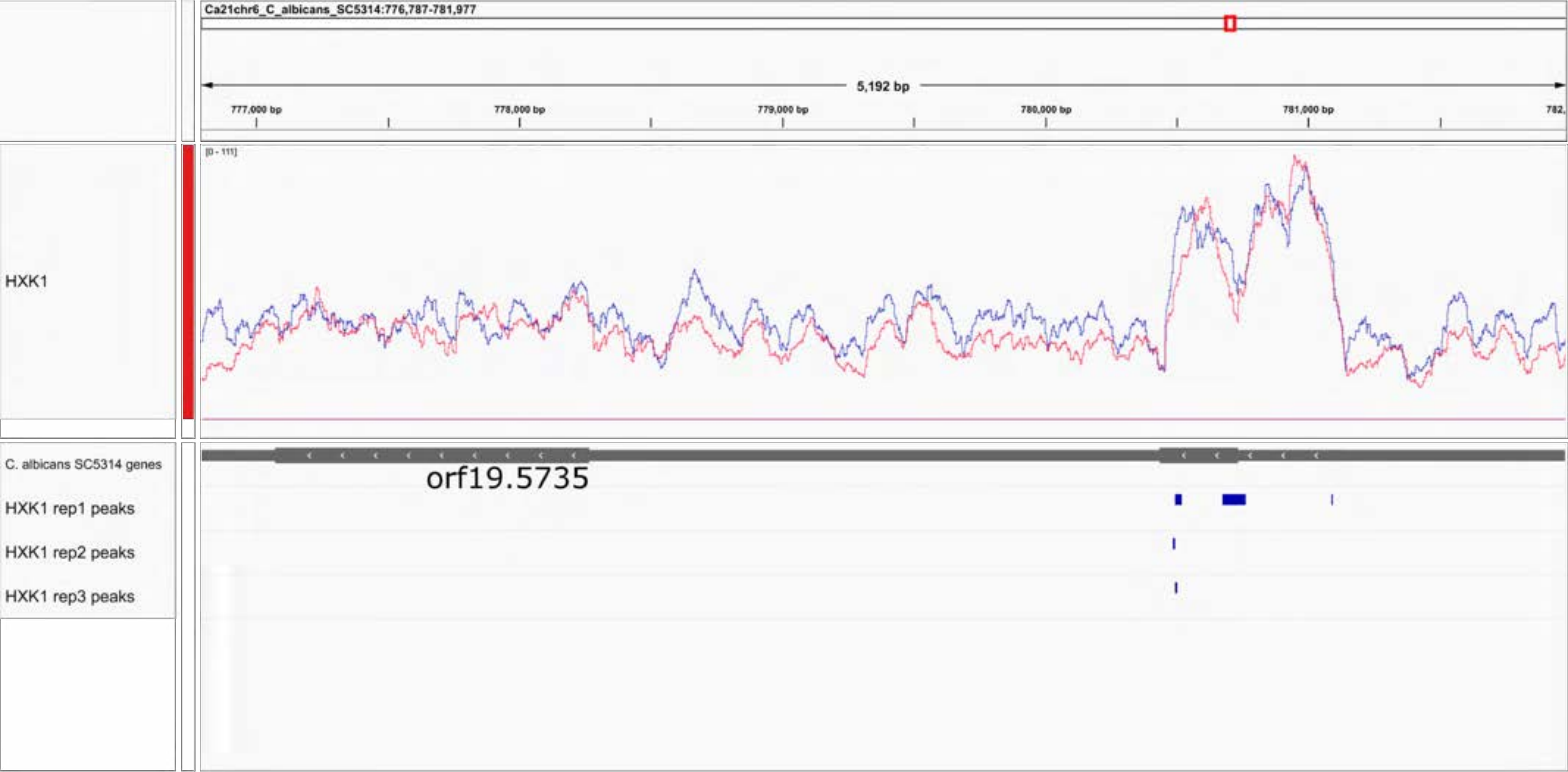
Ca21chr6_C_albicans_SC5314:776,787-781,977
5,192 bp
777,000 bp
778,000 bp
779,000 bp
780,000 bp
781,000 bp
HXK1
C. albicans SC5314 genes
orf19.5735
HXK1 rep1 peaks
HXK1 rep2 peaks
HXK1 rep3 peaks

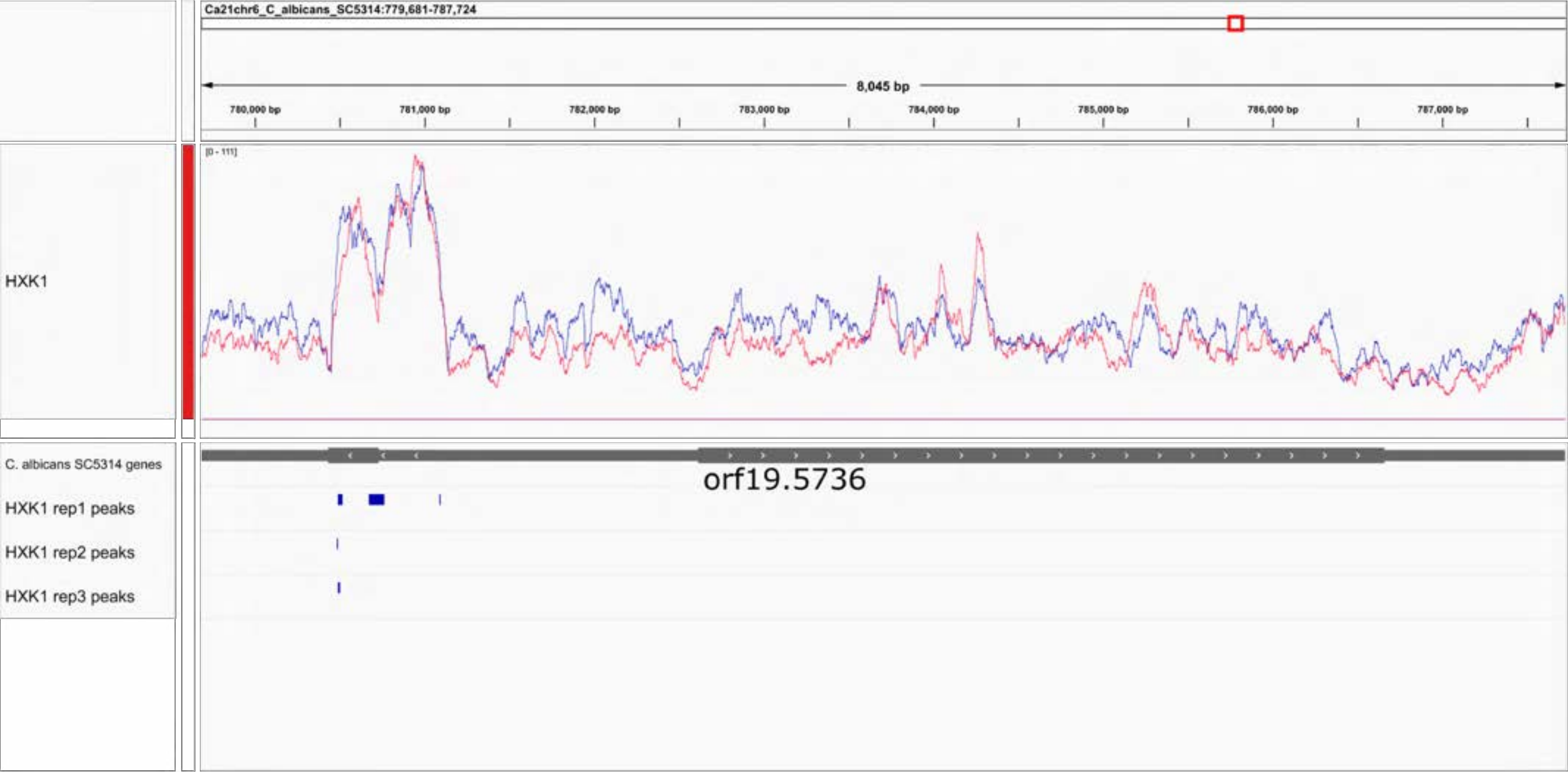

Ca21chr6_C_albicans_SC5314:779,681-787,724
8,045 bp
780,000 bp
781,000 bp
782,000 bp
783,000 bp
784,000 bp
785,000 bp
786,000 bp
787,000 bp
[0 - 111]
HXK1
C. albicans SC5314 genes
orf19.5736
HXK1 rep1 peaks
HXK1 rep2 peaks
HXK1 rep3 peaks

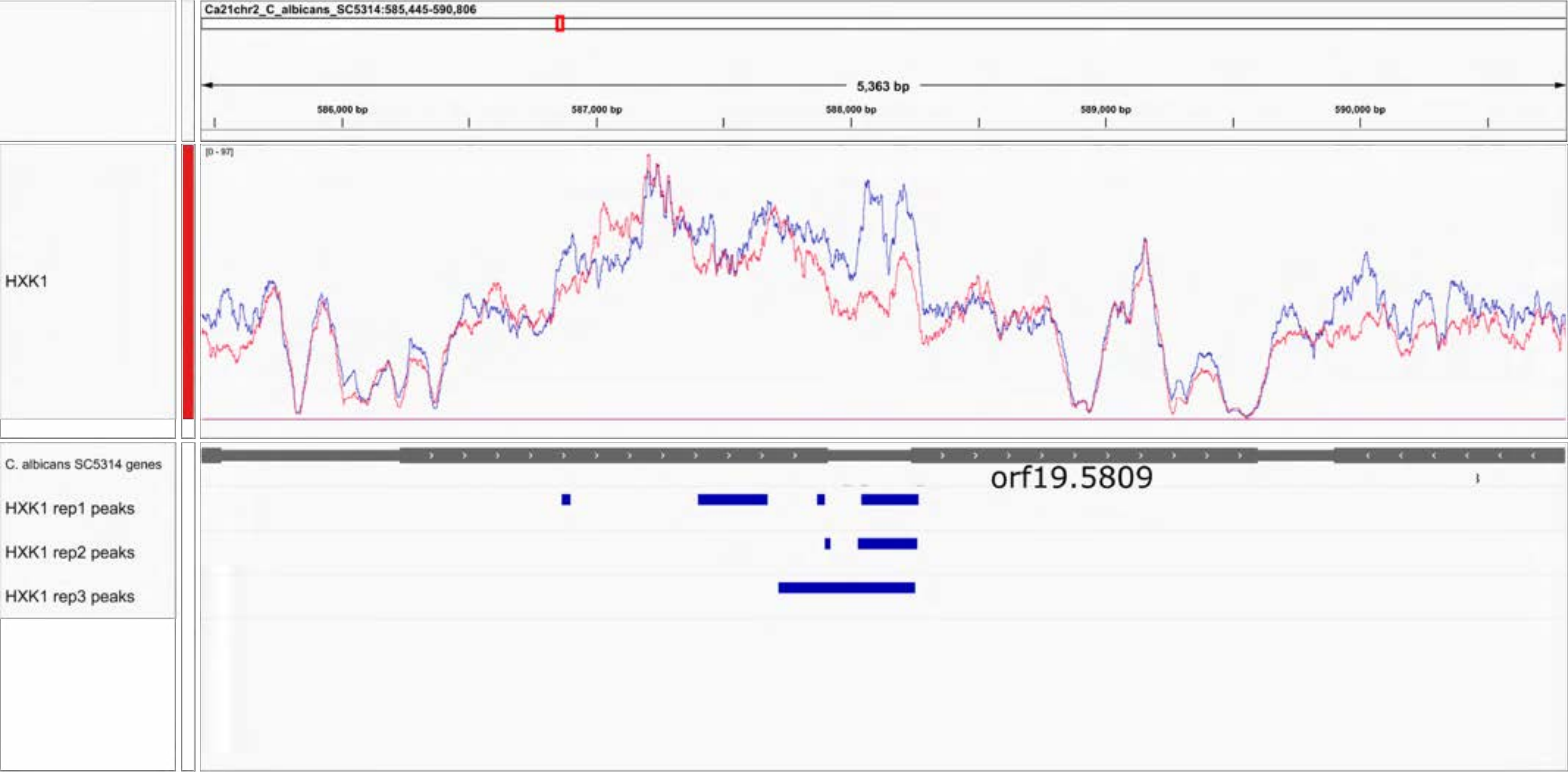

Ca21chr2_C_albicans_SC5314:585,445-590,806
5,363 bp
586,000 bp
587,000 bp
588,000 bp
589,000 bp
590,000 bp
HXK1
C. albicans SC5314 genes
orf19.5809
HXK1 rep1 peaks
HXK1 rep2 peaks
HXK1 rep3 peaks

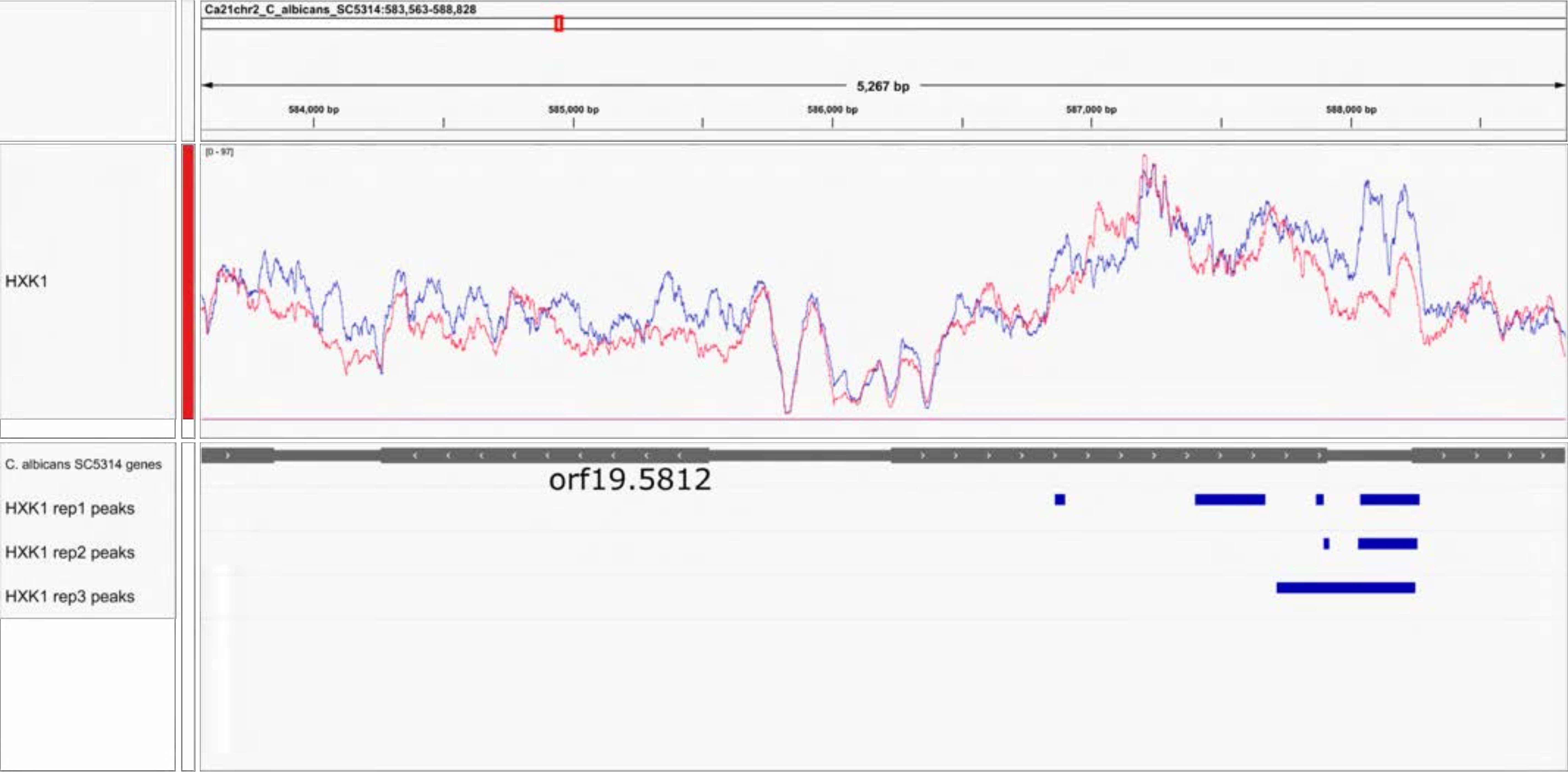

Ca21chr2_C_albicans_SC5314:583,563-588,828
5,267 bp
584,000 bp
585,000 bp
586,000 bp
587,000 bp
588,000 bp
HXK1
[0 - 97]
C. albicans SC5314 genes
orf19.5812
HXK1 rep1 peaks
HXK1 rep2 peaks
HXK1 rep3 peaks

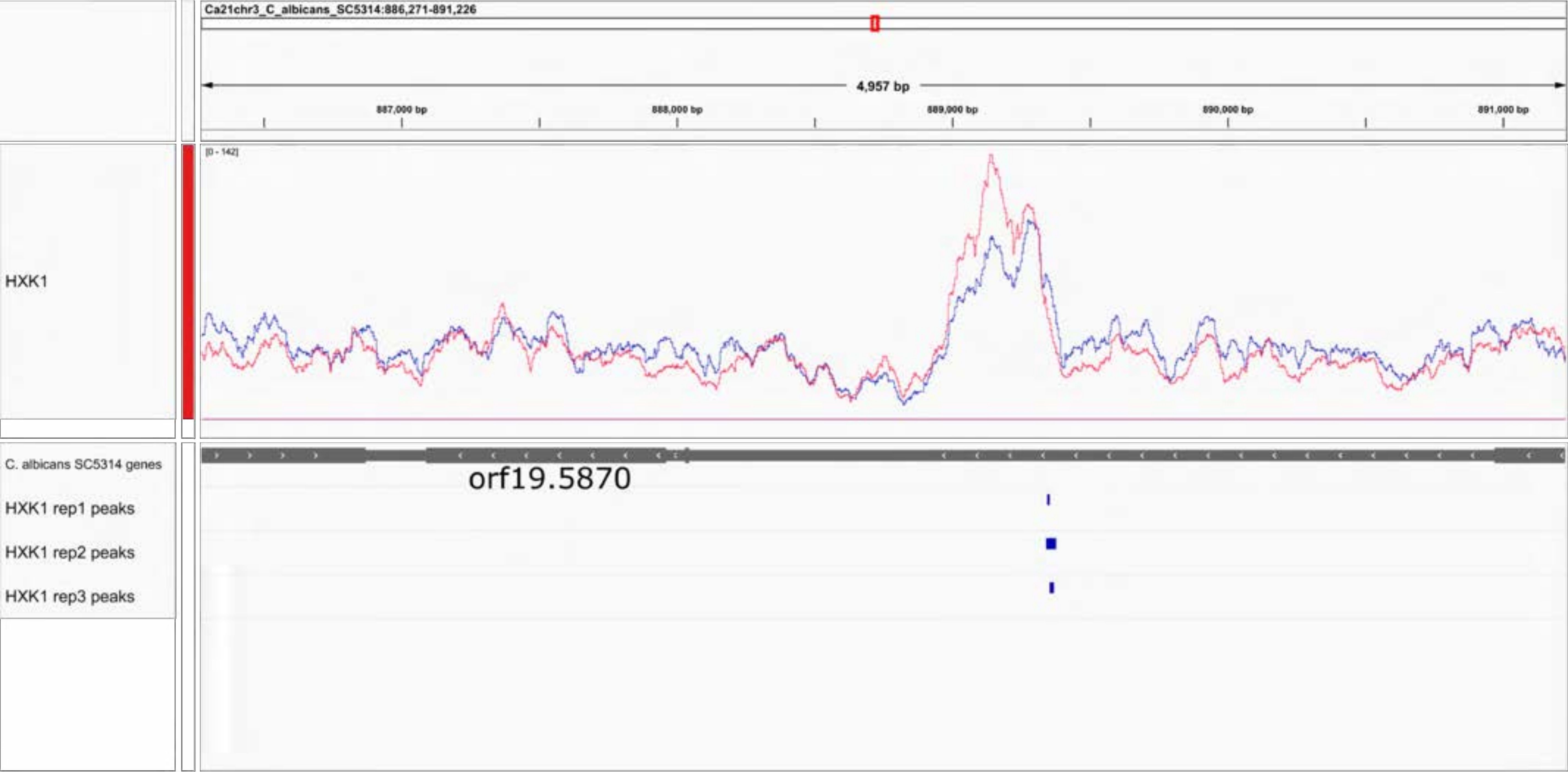
Ca21chr3_C_albicans_SC5314:886,271-891,226
4,957 bp
887,000 bp
888,000 bp
889,000 bp
890,000 bp
891,000 bp
HXK1
[0 - 142]
C. albicans SC5314 genes
orf19.5870
HXK1 rep1 peaks
HXK1 rep2 peaks
HXK1 rep3 peaks

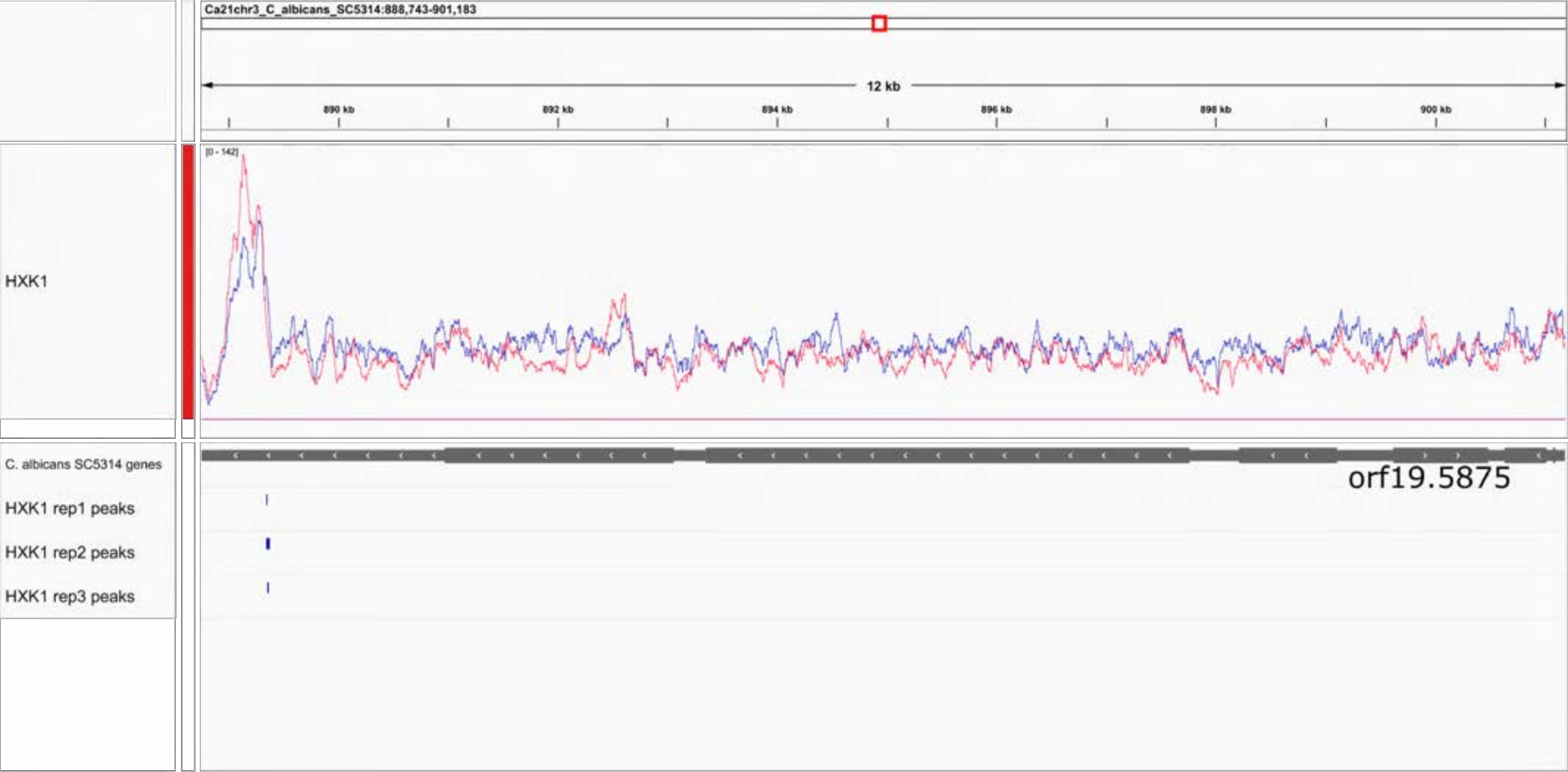

Ca21chr3_C_albicans_SC5314:888,743-901,183
12 kb
890 kb
892 kb
894 kb
896 kb
898 kb
900 kb
[0 - 142]
HXK1
C. albicans SC5314 genes
orf19.5875
HXK1 rep1 peaks
HXK1 rep2 peaks
HXK1 rep3 peaks

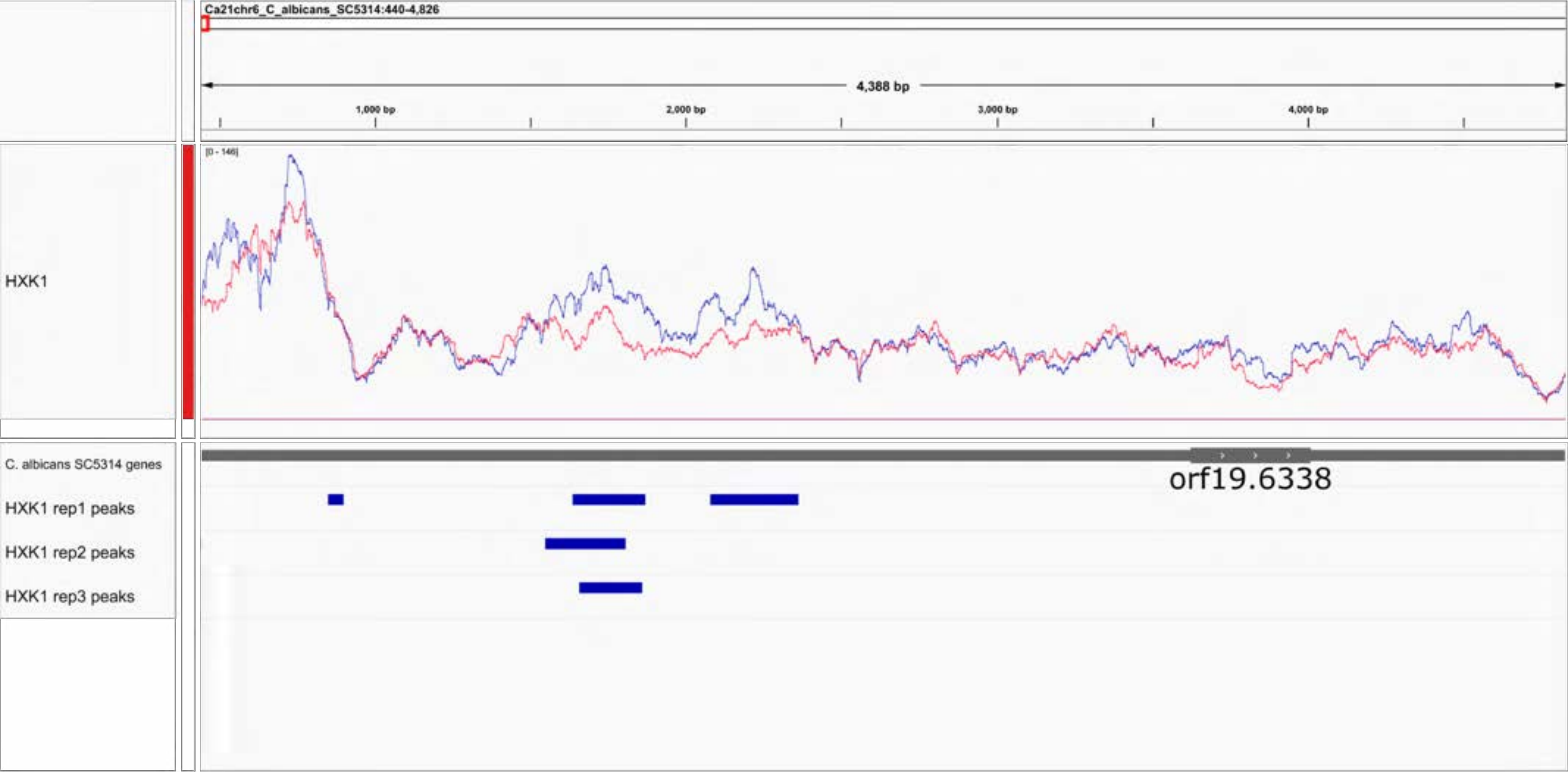

Ca21chr6_C_albicans_SC5314:440-4,826
4,388 bp
1,000 bp
2,000 bp
3,000 bp
4,000 bp
HXK1
[0 - 146]
C. albicans SC5314 genes
orf19.6338
HXK1 rep1 peaks
HXK1 rep2 peaks
HXK1 rep3 peaks

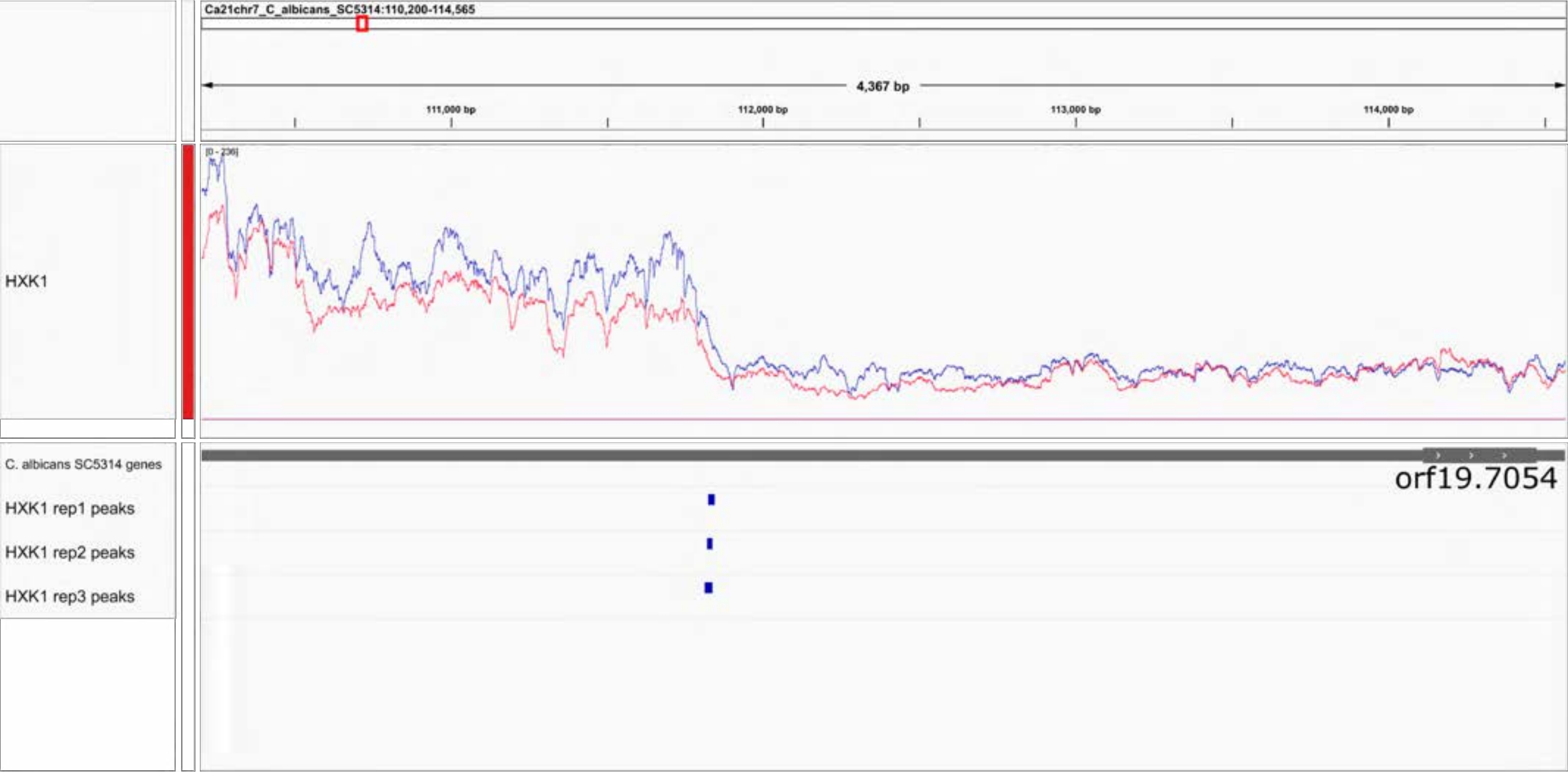
Ca21chr7_C_albicans_SC5314:110,200-114,565
4,367 bp
111,000 bp
112,000 bp
113,000 bp
114,000 bp
HXK1
C. albicans SC5314 genes
orf19.7054
HXK1 rep1 peaks
HXK1 rep2 peaks
HXK1 rep3 peaks

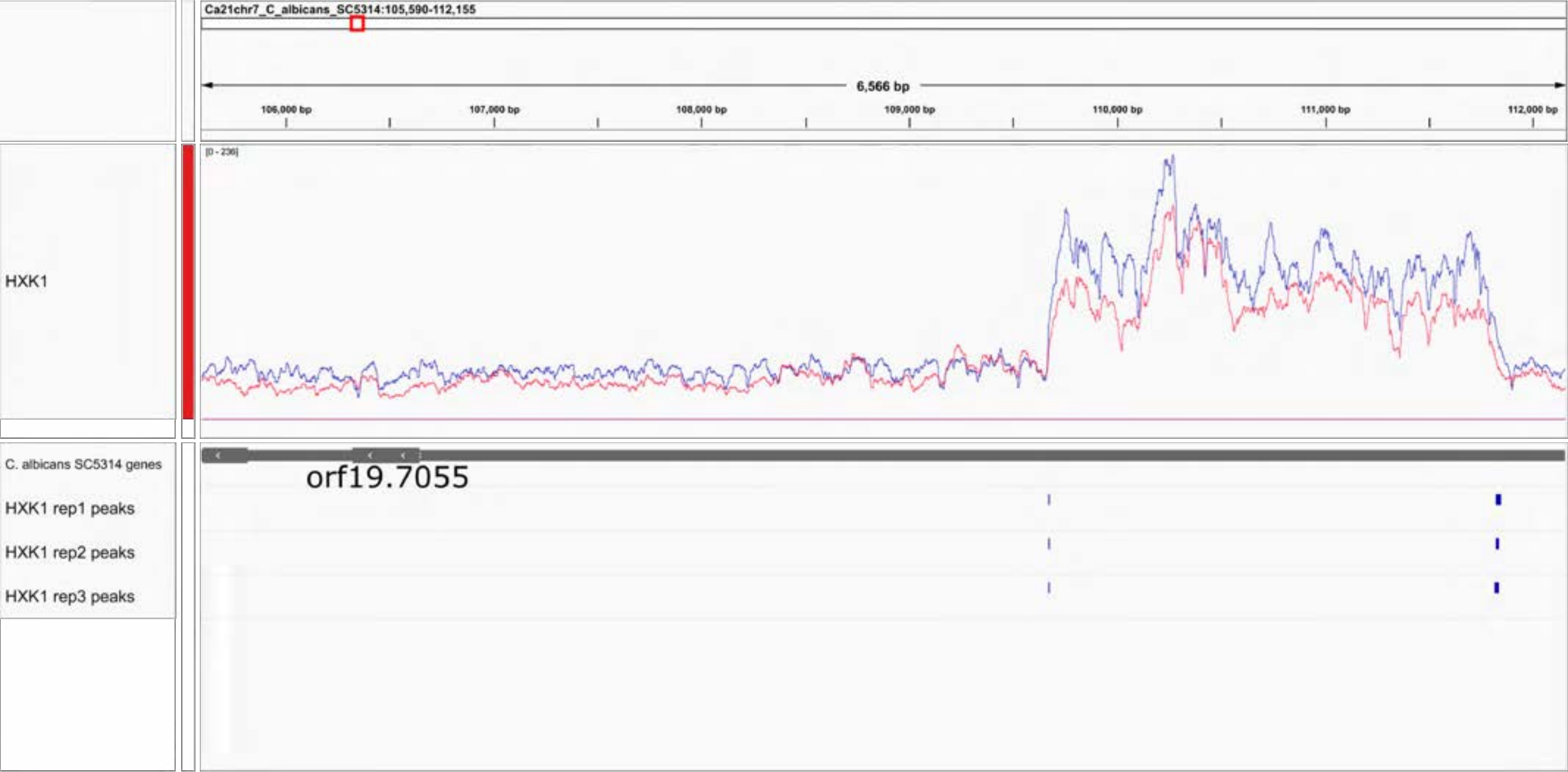

Ca21chr7_C_albicans_SC5314:105,590-112,155
6,566 bp
106,000 bp
107,000 bp
108,000 bp
109,000 bp
110,000 bp
111,000 bp
112,000 bp
HXK1
C. albicans SC5314 genes
orf19.7055
HXK1 rep1 peaks
HXK1 rep2 peaks
HXK1 rep3 peaks

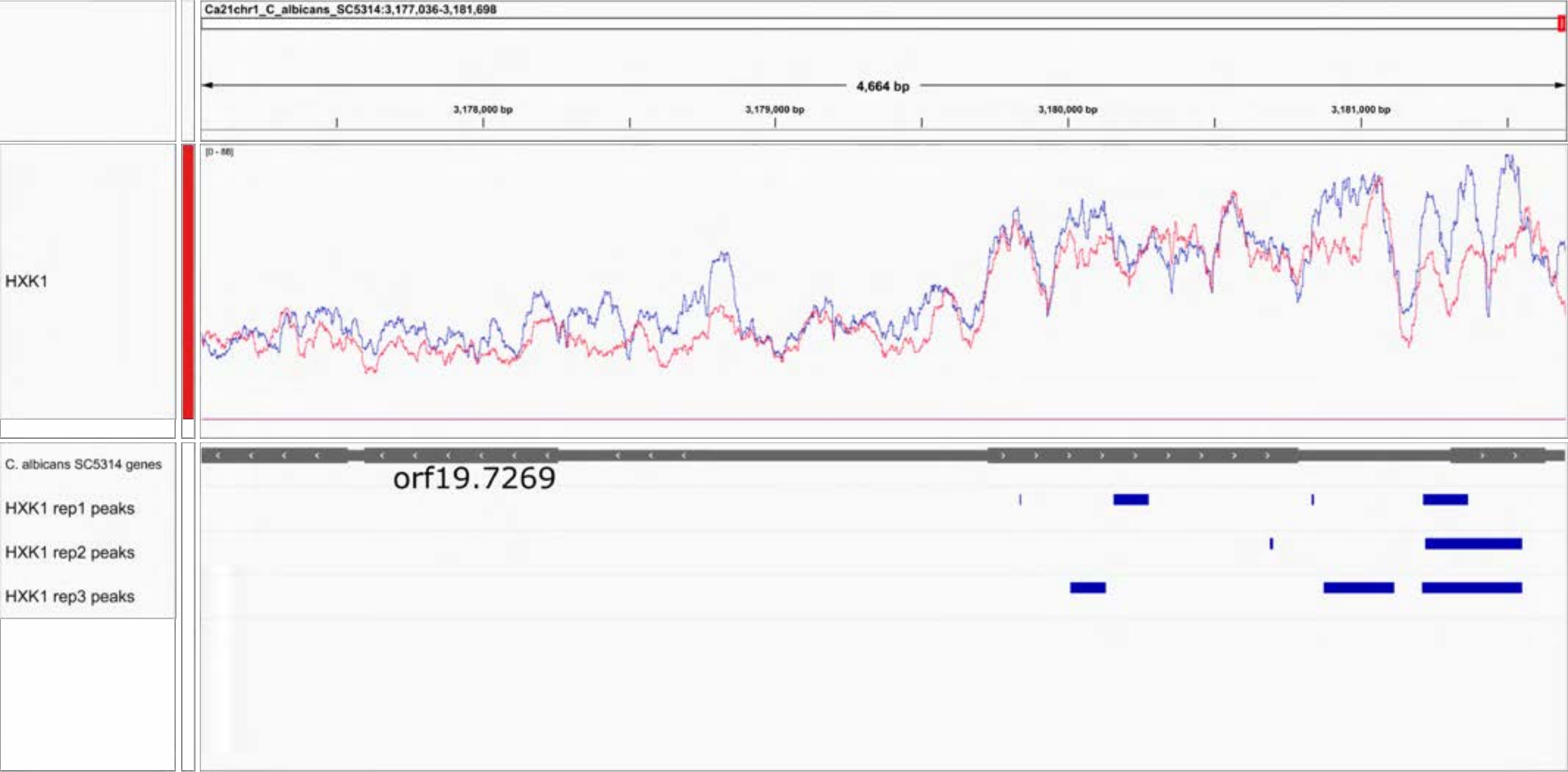

Ca21chr1_C_albicans_SC5314:3,177,036-3,181,698
4,664 bp
3,178,000 bp
3,179,000 bp
3,180,000 bp
3,181,000 bp
HXK1
[0 - 88]
C. albicans SC5314 genes
orf19.7269
HXK1 rep1 peaks
HXK1 rep2 peaks
HXK1 rep3 peaks

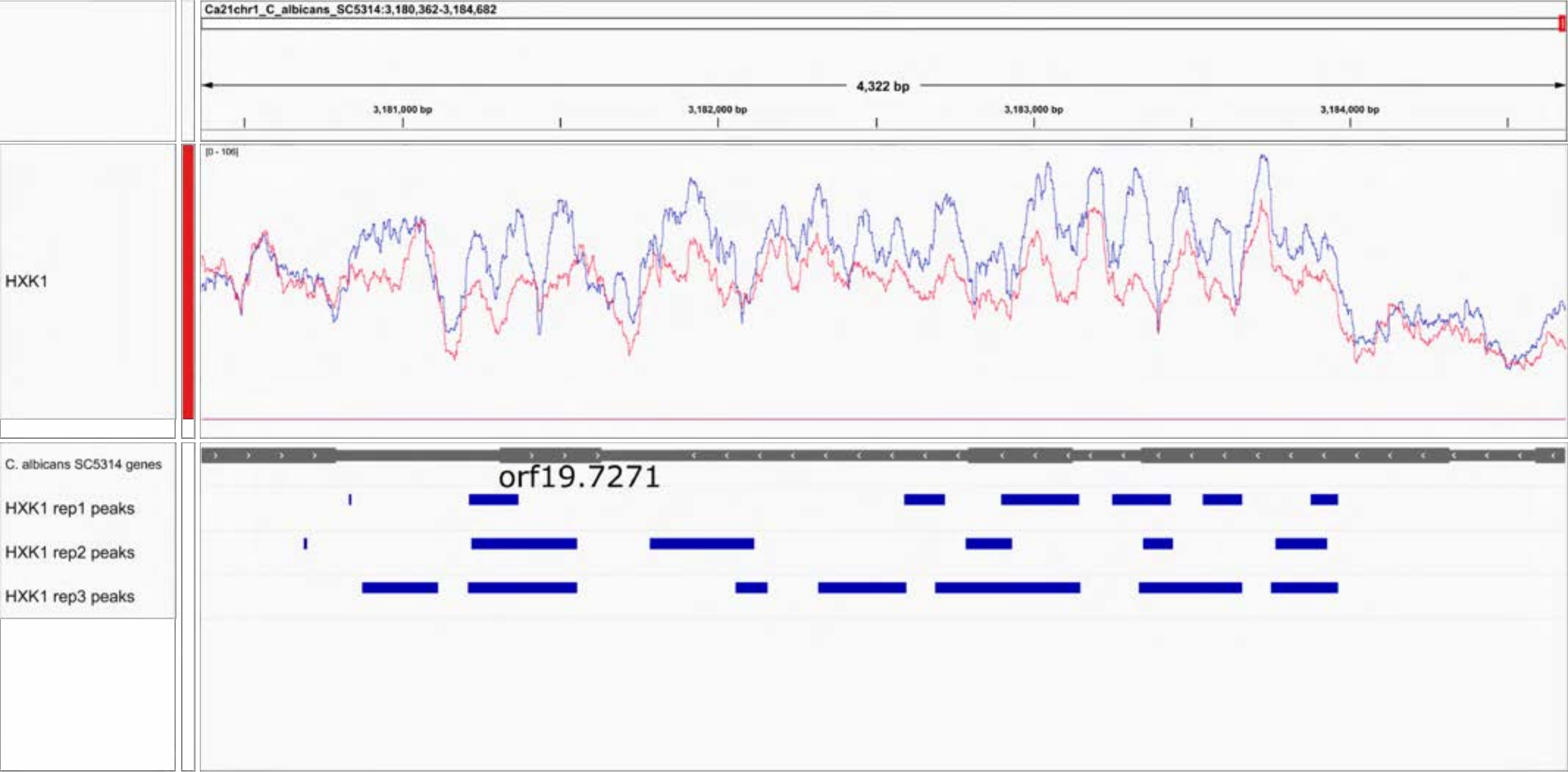

Ca21chr1_C_albicans_SC5314:3,180,362-3,184,682
4,322 bp
3,181,000 bp
3,182,000 bp
3,183,000 bp
3,184,000 bp
HXK1
[0 - 106]
C. albicans SC5314 genes
orf19.7271
HXK1 rep1 peaks
HXK1 rep2 peaks
HXK1 rep3 peaks

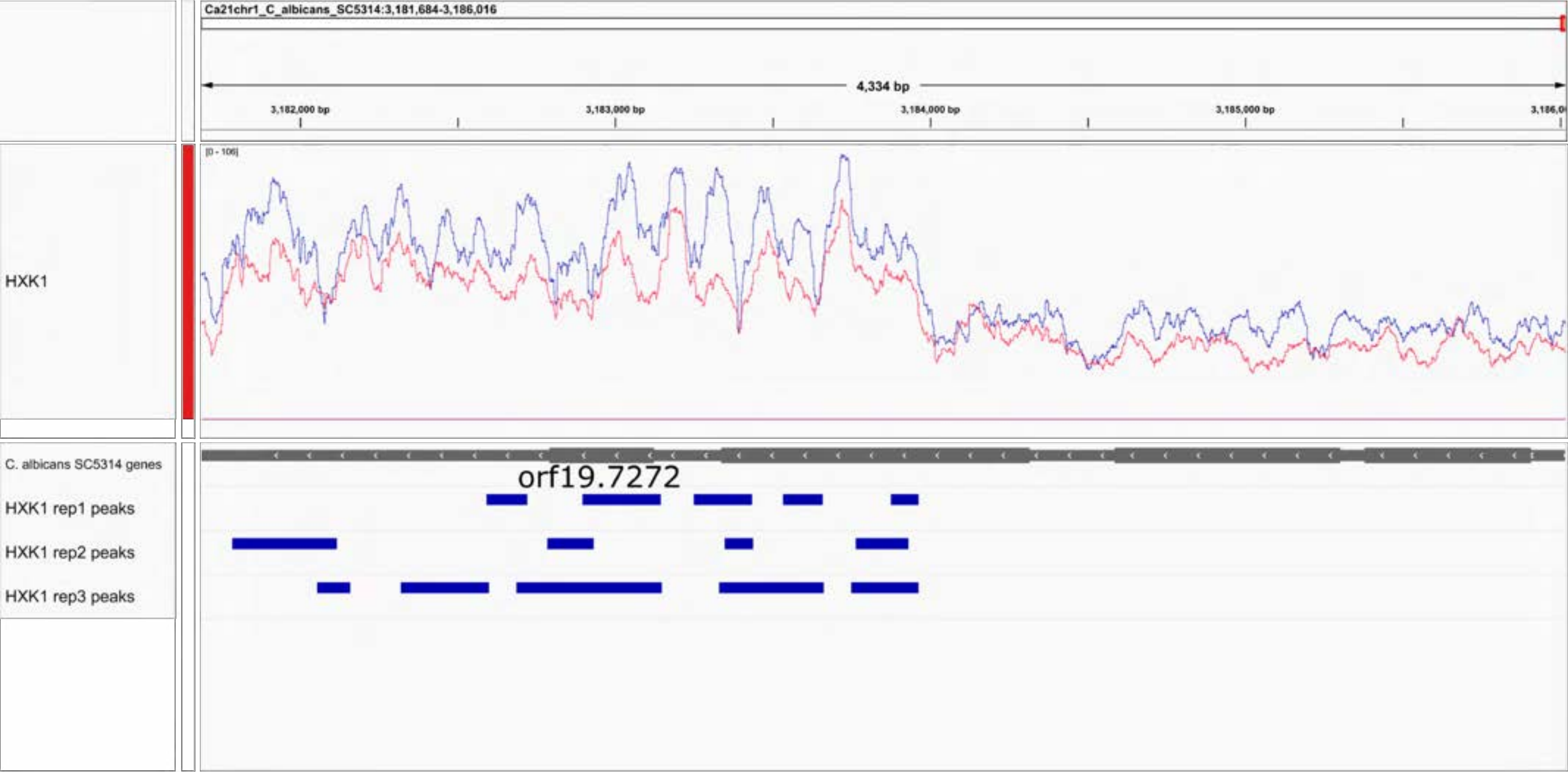
Ca21chr1_C_albicans_SC5314:3,181,684-3,186,016
4,334 bp
3,182,000 bp
3,183,000 bp
3,184,000 bp
3,185,000 bp
HXK1
[0 - 106]
C. albicans SC5314 genes
orf19.7272
HXK1 rep1 peaks
HXK1 rep2 peaks
HXK1 rep3 peaks

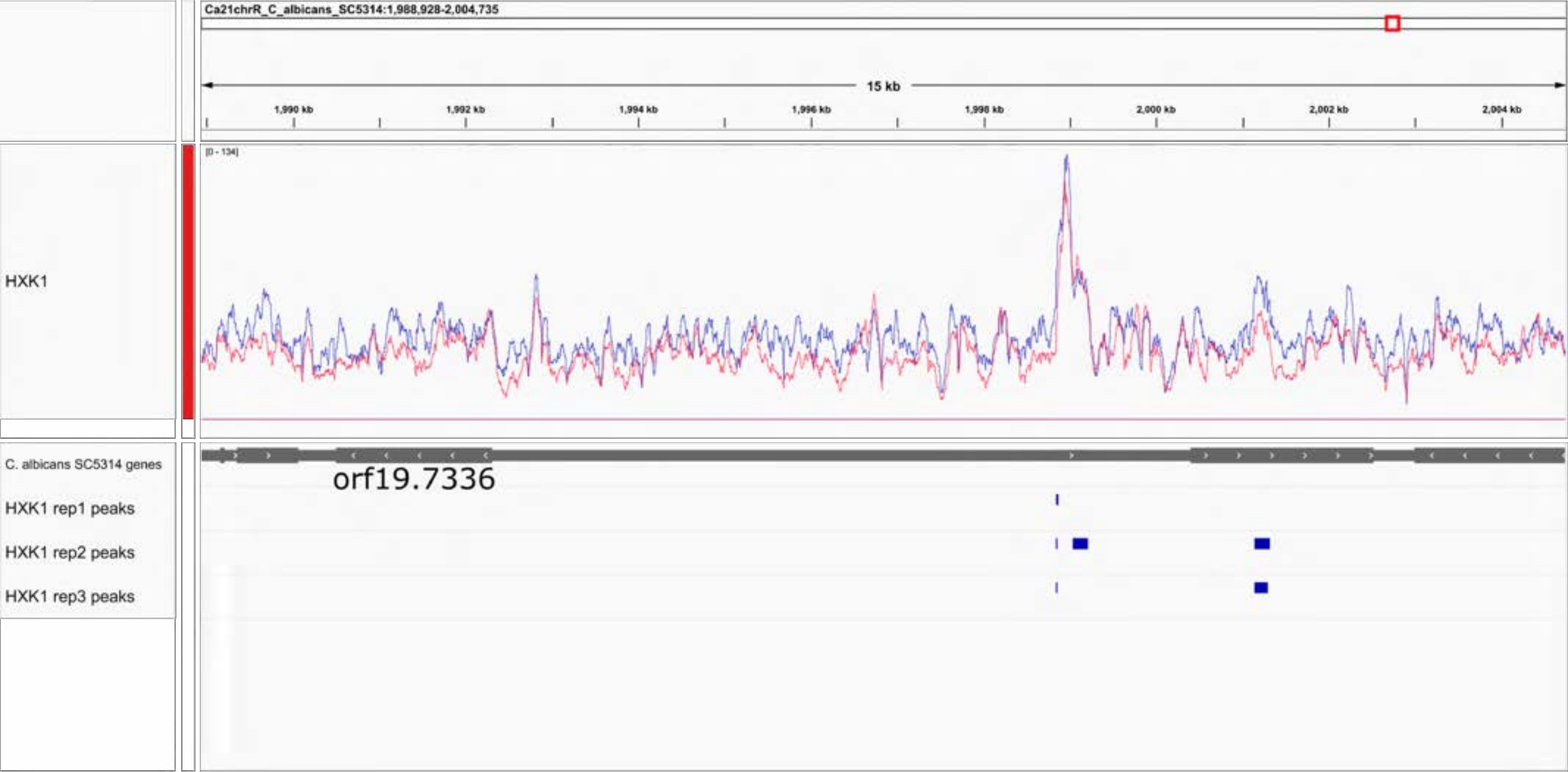

Ca21chrR_C_albicans_SC5314:1,988,928-2,004,735
15 kb
1,990 kb
1,992 kb
1,994 kb
1,996 kb
1,998 kb
2,000 kb
2,002 kb
2,004 kb
HXK1
[0 - 134]
C. albicans SC5314 genes
orf19.7336
HXK1 rep1 peaks
HXK1 rep2 peaks
HXK1 rep3 peaks

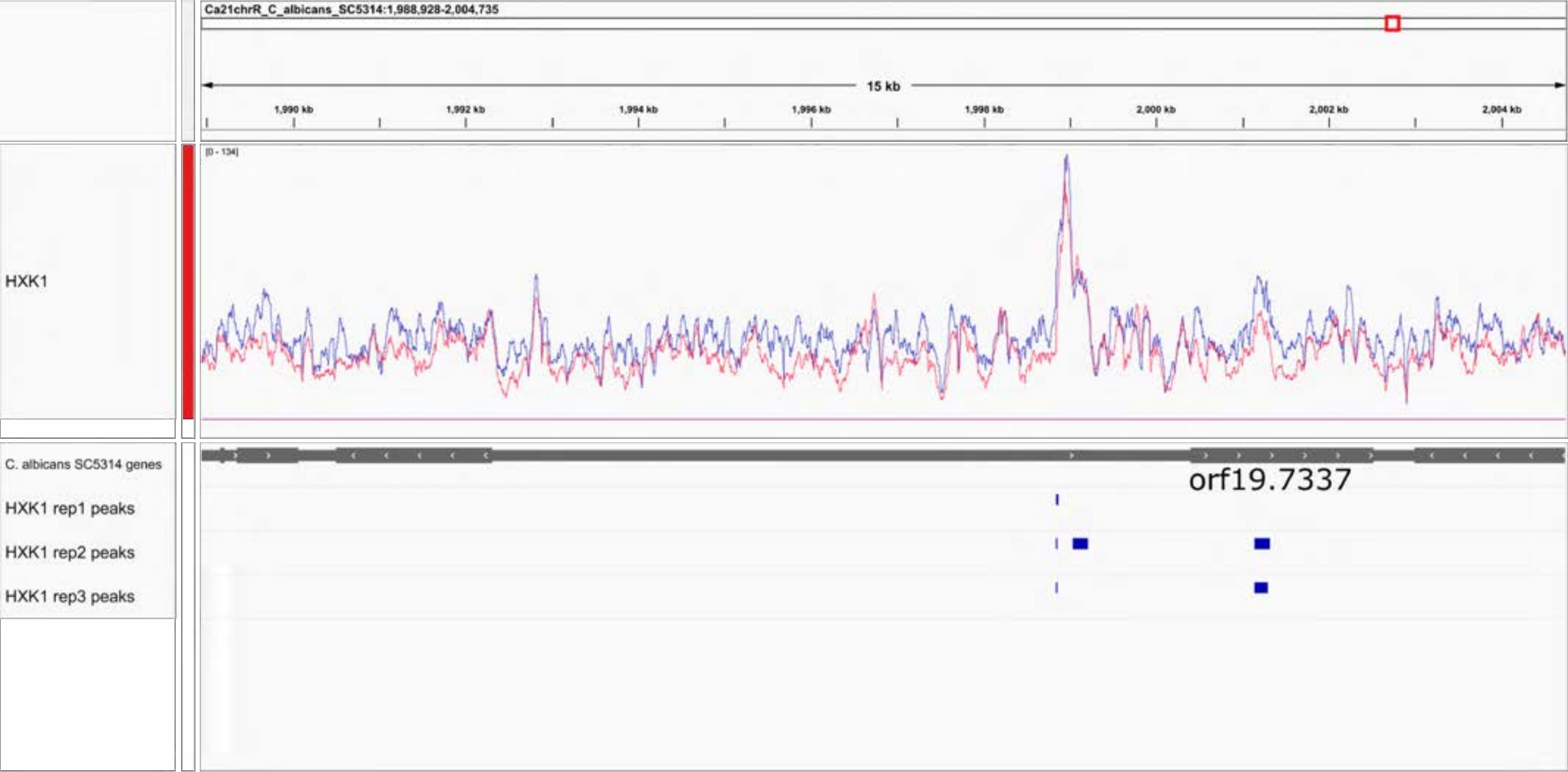
Ca21chrR_C_albicans_SC5314:1,988,928-2,004,735
15 kb
1,990 kb
1,992 kb
1,994 kb
1,996 kb
1,998 kb
2,000 kb
2,002 kb
2,004 kb
HXK1
[0 - 134]
C. albicans SC5314 genes
orf19.7337
HXK1 rep1 peaks
HXK1 rep2 peaks
HXK1 rep3 peaks

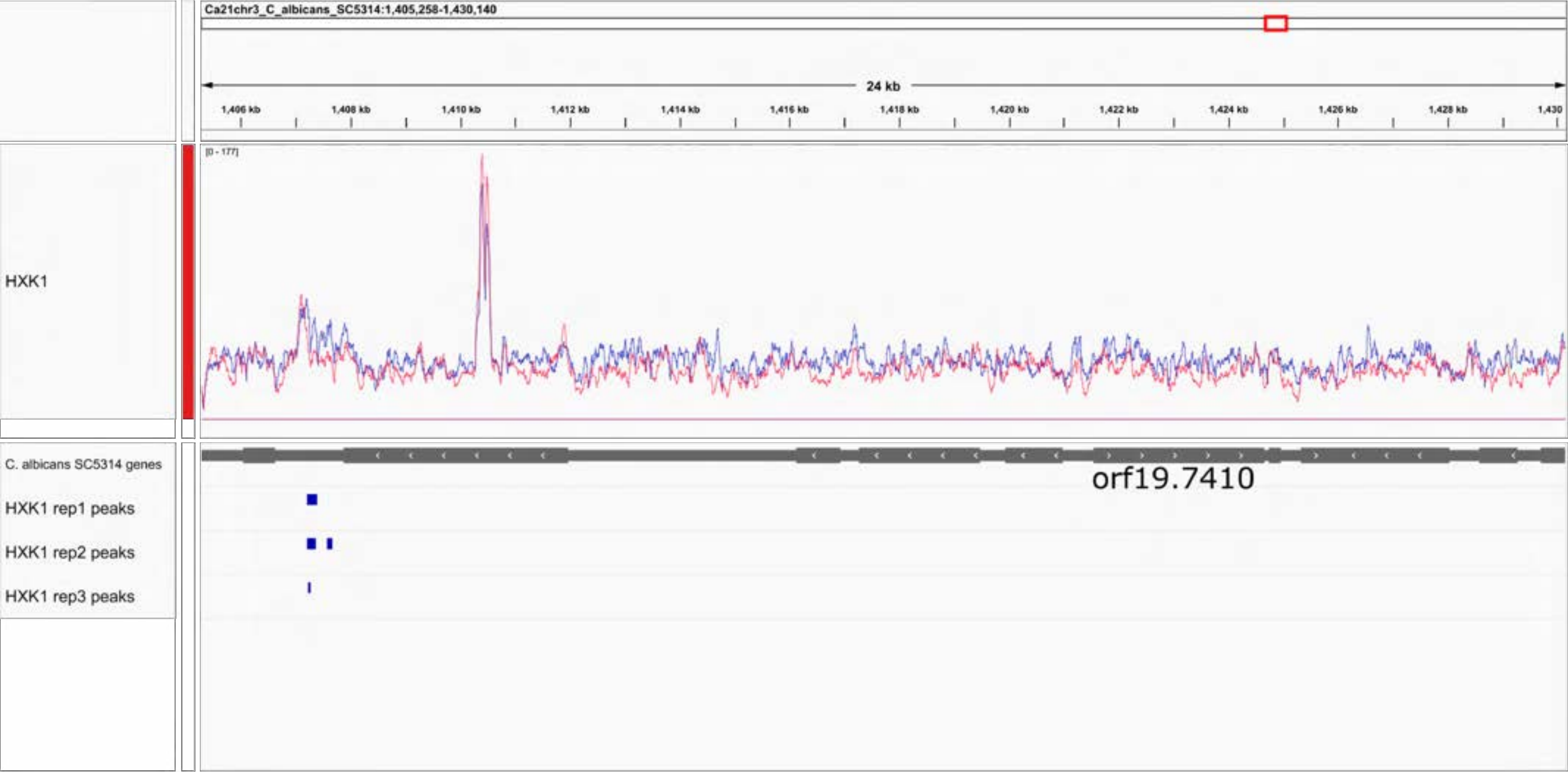
Ca21chr3_C_albicans_SC5314:1,405,258-1,430,140
24 kb
1,406 kb
1,408 kb
1,410 kb
1,412 kb
1,414 kb
1,416 kb
1,418 kb
1,420 kb
1,422 kb
1,424 kb
1,426 kb
1,428 kb
1,430
HXK1
[0 - 177]
C. albicans SC5314 genes
orf19.7410
HXK1 rep1 peaks
HXK1 rep2 peaks
HXK1 rep3 peaks

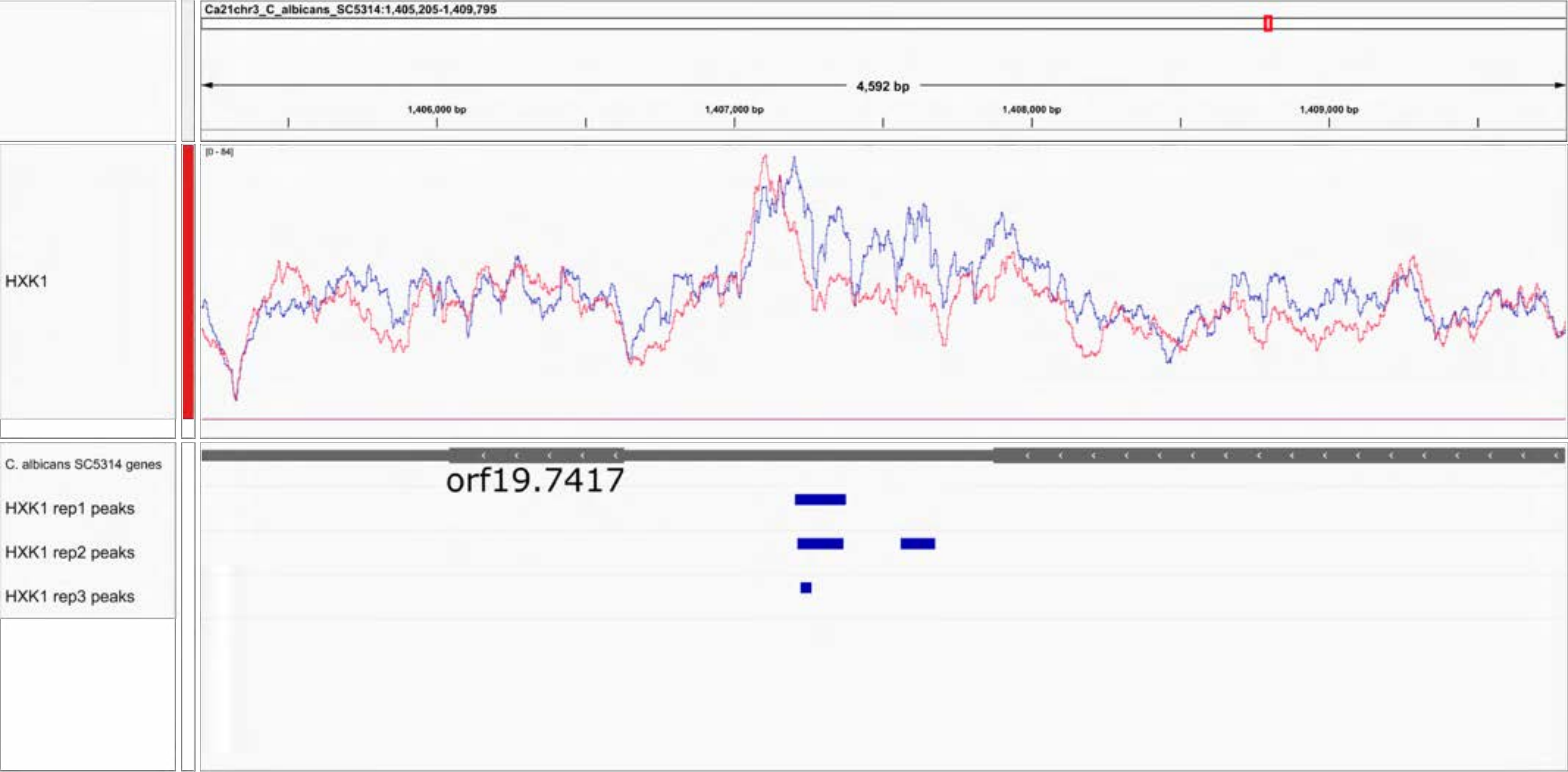
Ca21chr3_C_albicans_SC5314:1,405,205-1,409,795
4,592 bp
1,406,000 bp
1,407,000 bp
1,408,000 bp
1,409,000 bp
HXK1
C. albicans SC5314 genes
orf19.7417
HXK1 rep1 peaks
HXK1 rep2 peaks
HXK1 rep3 peaks
