# Supplementary figures and images for: Candida albicans Hxk1 influences expression of metabolic- and virulence-related genes
Source: mSphere. 2025 Sep 25;10(10):e00395-25. doi: 10.1128/msphere.00395-25 (PMC12570500; doi:10.1128/msphere.00395-25)

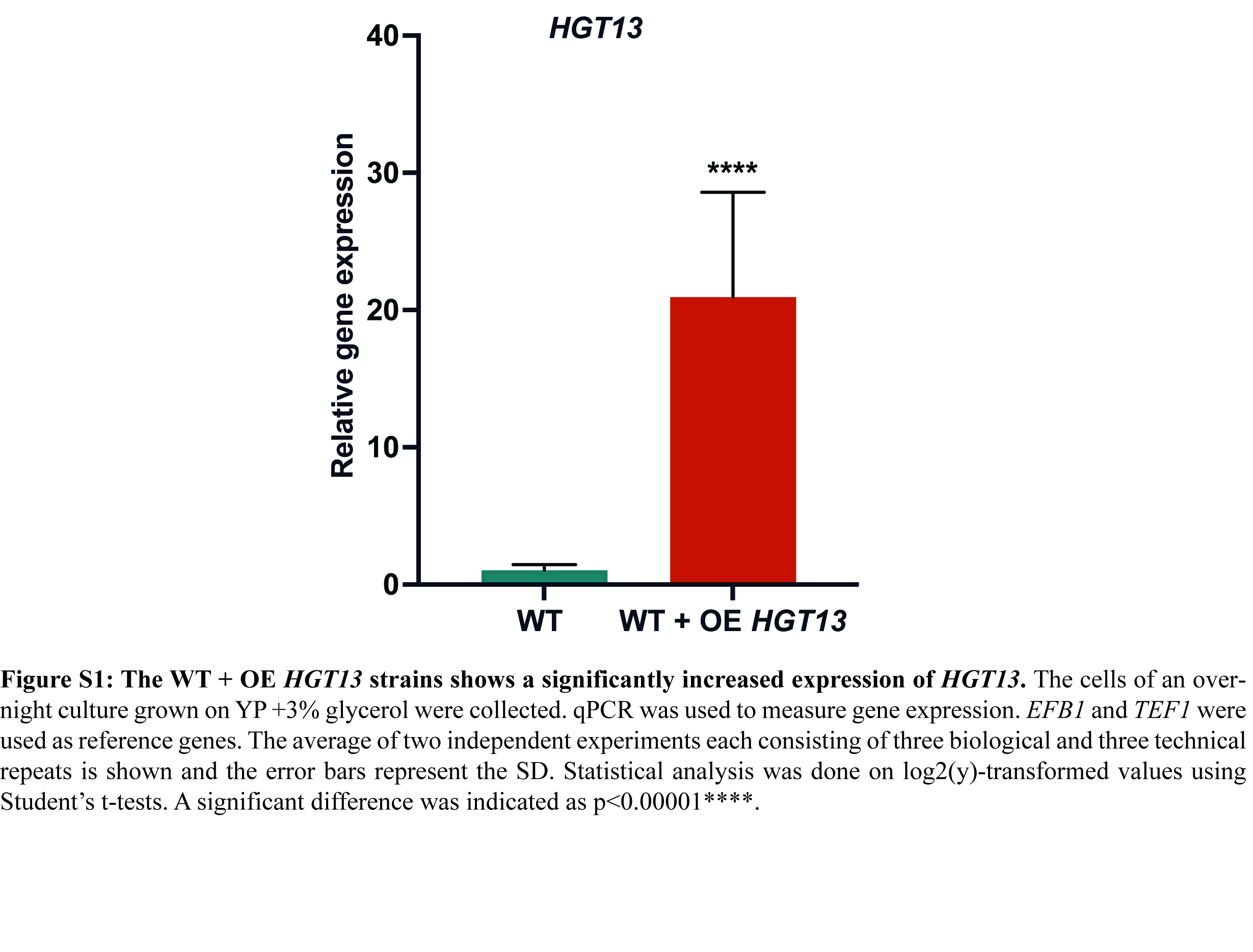

Supplement: Figure S1 — The expression of HGT13 was measured in an HGT13 overexpression strain. [file msphere.00395-25-s0005.tif]
