# Supplementary material for: Candida albicans Hxk1 influences expression of metabolic- and virulence-related genes
Source: mSphere. 2025 Sep 25;10(10):e00395-25. doi: 10.1128/msphere.00395-25 (PMC12570500; doi:10.1128/msphere.00395-25)
Supplement: Table S1 — qPCR primers used in this study. [file msphere.00395-25-s0006.docx]

| **Gene** | **Primers** | |
| --- | --- | --- |
|  | **Forward** | **Reverse** |
| *18S* | GATGCCCTTAGACGTTCTGG | CACGACGGAGTTTCACAAGA |
| *ACT1* | TGTTGGTGATGAAGCCCAATC | CATATCGTCCCAGTTGGAAACAA |
| *EFB1* | GCTGCCAAATCTATTGTCACCT | TGAGCACCCCAAGTCAAAC |
| *TEF1* | CCACTGAAGTCAAGTCCGTTGA | CACCTTCAGCCAATTGTTCGT |
| *DAC1* | GAGGAGTCTACCGCAGAGGA | GGACACGTAGCAGTAACCCC |
| *NAG1* | TTTGGCATTCAACGAAGCGG | GTCGTTCCCGAAAAACCTGC |
| *HEX1* | AGGATTCCAACGGACACCAC | CACCAAAGGAGAAACCCCGT |
| *NGT1* | GGGTATTGGGAGCCATGTCC | TCTCCAAGCAATAGCAGCCC |
| *GAL1* | GCGAGTGGTAGAGATGGCAA | ACCCCTCTTAAACTGCTAGTGC |
| *GAL7* | TGGTAACATACGTGCTACTGGT | TGATCCAACCTCACAGCAGG |
| *GAL10* | GGTCCAGTTGTGAAAAGTCGTG | TTCAAGCTCACCTGGGAACC |
| *GAL102* | AGTTCATGGCAAGGGAACCA | CATGGGGTTTTTGATCCACACA |
| *HGT13* | AGAGCAGAAAACGGAGGTGG | TGTGCCTCCCAAATACAGCA |
| *SOD4* | AGCTTACATTGGTGGGTTGTCT | AGCAGCACTTGCAGTATCGT |
| *SOD5* | GGACACGGCAATGCTAACAC | CCTTGAGGAGCAGTAGAAGCC |
| *ECE1* | CCGTCGTCAGATTGCCAGAA | ACATCTGGAACGCCATCTCTC |
| *HWP1* | GCTGGCTCAAGTGGTGCTAT | GCAGATGGTTGCATGAGTGG |
| *LIP1* | AACGCTGACCCATCCAAAGT | GTTGACCAAGGGGCACCATA |
| *PRY1* | TGGTTTTGGATCTGGATTTGGC | TGAGCTTGAAGGTTGTTGTTGG |

**Table S1 qPCR primers**
